# Fast Coding of Orientation in Primary Visual Cortex

**DOI:** 10.1371/journal.pcbi.1002536

**Published:** 2012-06-14

**Authors:** Oren Shriki, Adam Kohn, Maoz Shamir

**Affiliations:** 1 Department of Physiology and Neurobiology, Ben-Gurion University of the Negev, Be'er-Sheva, Israel; 2 Laboratory of Systems Neuroscience, National Institute of Mental Health, Bethesda, Maryland, United States of America; 3 Dom Purpura Department of Neuroscience, Albert Einstein College of Medicine, New York, New York, United States of America; 4 Department of Ophthalmology and Visual Sciences, Albert Einstein College of Medicine, New York, New York, United States of America; 5 Department of Physics, Ben-Gurion University of the Negev, Be'er-Sheva, Israel; Université Paris Descartes, Centre National de la Recherche Scientifique, France

## Abstract

Understanding how populations of neurons encode sensory information is a major goal of systems neuroscience. Attempts to answer this question have focused on responses measured over several hundred milliseconds, a duration much longer than that frequently used by animals to make decisions about the environment. How reliably sensory information is encoded on briefer time scales, and how best to extract this information, is unknown. Although it has been proposed that neuronal response latency provides a major cue for fast decisions in the visual system, this hypothesis has not been tested systematically and in a quantitative manner. Here we use a simple ‘race to threshold’ readout mechanism to quantify the information content of spike time latency of primary visual (V1) cortical cells to stimulus orientation. We find that many V1 cells show pronounced tuning of their spike latency to stimulus orientation and that almost as much information can be extracted from spike latencies as from firing rates measured over much longer durations. To extract this information, stimulus onset must be estimated accurately. We show that the responses of cells with weak tuning of spike latency can provide a reliable onset detector. We find that spike latency information can be pooled from a large neuronal population, provided that the decision threshold is scaled linearly with the population size, yielding a processing time of the order of a few tens of milliseconds. Our results provide a novel mechanism for extracting information from neuronal populations over the very brief time scales in which behavioral judgments must sometimes be made.

## Author Summary

How can humans and animals make complex decisions on time scales as short as 100 ms? The information required for such decisions is coded in neural activity and should be read out on a very brief time scale. Traditional approaches to coding of neural information rely on the number of electrical pulses, or spikes, that neurons fire in a certain time window. Although this type of code is likely to be used by the brain for higher cognitive tasks, it may be too slow for fast decisions. Here, we explore an alternative code which is based on the latency of spikes with respect to a reference signal. By analyzing the simultaneous responses of many cells in monkey visual cortex, we show that information about the orientation of visual stimuli can be extracted reliably from spike latencies on very short time scales.

## Introduction

Firing rates of many primary visual cortical cells are tuned to the orientation of visual stimuli [1]. This dependence of neuronal firing rates on the stimulus implies that information about the stimulus can be decoded from the spike count. The trial to trial variability of firing limits the accuracy with which a stimulus can be estimated from the neuronal spike count [2]–[4]. To decrease this variability and increase the accuracy of the rate code, studies have typically used responses measured over several hundred milliseconds [1], [2], [5]. However, increasing evidence indicates that the central nervous system can process complex information on very short time scales.

Visual psychophysical and evoked potential studies have shown that human subjects can classify natural scenes or emotional facial expressions on the basis of 100–150 ms of processing [6]–[12]. Evidence for fast processing of visual stimuli also exists from behavioral and electrophysiological experiments in monkeys [13]–[15]. A recent study by Stanford et al. [15] shows that monkeys can make perceptual decisions regarding the color of stimuli after about 30 ms of processing time. Evidence for fast coding also exists for the auditory system [16], [17] and the somatosensory system [18], [19]. The overall theme deriving from these studies is that sensory systems are able to process the gist of a scene rapidly [20].

It has been suggested that the temporal structure of the neuronal response and in particular, response latency, is the source of fast decisions in the brain [18], [19], [21]–[33]. However, the accuracy of codes based on these responses has not been studied in the visual system systematically.

A common approach to measuring response latency is to define it as the transition from spontaneous firing to *stimulus-dependent* firing, e.g., by detecting the time at the which the PSTH (Post Stimulus Time Histogram) reaches half of its maximal firing rate [26]. This attempts to estimate the ‘pure’ latency component of the response, but it involves defining that quantity by a different number of spikes for each condition. For instance, latency might be defined by the time to the first ten spikes at the preferred stimulus and to the first spike at a non-preferred stimulus. Thus, in this approach the criterion for neural response time depends on the stimulus, making it impractical for decoding: the readout parameters cannot scale in a stimulus dependent manner, as that requires the readout to know the stimulus in order to estimate it.

Recently, we proposed a simple spike latency code readout [34], the temporal Winner-take-all (tWTA). The tWTA determines the external stimulus by the label, e.g. preferred orientation, of the cell that fired the first spike in the population. It avoids attempting to estimate ‘pure’ onset latency and instead takes a pragmatic approach in which each cell's time to first spike will depend both on its latency and the strength of its response.

Formally, consider a population of *N* neurons coding for the orientation of a visual stimulus, 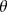. Let us denote by 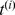 the time of the first spike of neuron *i*, with preferred orientation 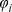, following some reference signal *t*_ref_. The tWTA algorithm estimates the orientation of the external stimulus as the preferred orientation of the neuron which fired *first* with respect to *t*_ref_:
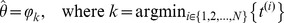
This definition can be generalized to estimate the stimulus by the preferred orientation of the cell that fired the *n*th spike first, or to incorporate a competition between groups, ‘columns’ of cells (see below). Here we investigate neural coding on brief time scales by applying the tWTA to simultaneously recorded populations of neurons in the primary visual cortex of macaque monkeys responding to the orientation of visual stimuli.

## Results

Responses of multiple neurons were measured in primary visual cortex (V1) of anesthetized monkeys using electrode arrays. The stimuli were drifting sinusoidal gratings of varying orientations. The duration of each stimulus was 300–400 ms and each stimulus was repeated 200–400 times. Details about stimulus parameters and numbers of recorded units in each dataset appear in Table 1 (see Materials and Methods). The recorded cells consisted of well-isolated single units and small multiunit clusters.

**Table 1:** Detailed description of datasets.

Dataset	Stimulus type	# of Orientations	Contrast (%)	Size (deg)	Spatial freq. (cycles/deg)	Temporal freq. (cycles/sec)	Initial phase	Stimulus duration (ms)	Inter Stimulus Interval (ms)	# of Repetitions	# of units	# of tuned units	# of onset detectors
1	DG^a^	8	100	2×2.9^b^	1	6.25	Fix^c^	400	800	400	111	54	28
2	DG	8	100	2×2.9	1	6.25	Fix	400	800	400	84	48	18
3	DG	36	100	5.2	1	3	Fix	300	500	200	159	69	25
4	DG	8	100	2.75	1	3	Fix	300	500	300	158	74	10
5	DG	8	100	2.75	1	3	Ran^d^	300	500	300	130	68	13
6	SG^e^	8	100	2×3.5	1	NA^f^	Fix	50	500	300	153	89	26
								300				98	10

a DG – Drifting grating.

b 2x – 2 spatially offset gratings were presented, of the indicated size. This was done when the direction of gaze of the two eyes was different. Each grating covered the receptive fields in one eye.

c Fix – Fixed.

d Ran – Random.

e SG – Static grating.

f NA – Not applicable.

The table describes the stimulus parameters for each dataset as well the total number of simultaneously recorded units after spike sorting (where units refer to both single units and multiunit activity consisting of small clusters of cells), the number of tuned cells and the number of onset detectors. The number of tuned cells corresponds to the number of cells for which the modulation amplitude of the first spike latency tuning curve, *B*, was larger than 15 ms for all stimulus conditions. The number of onset detectors corresponds to the cells that had both B<15 ms and spontaneous rate smaller than 5 spks/sec.

### Tuning of spike latencies

**Figure 1:**
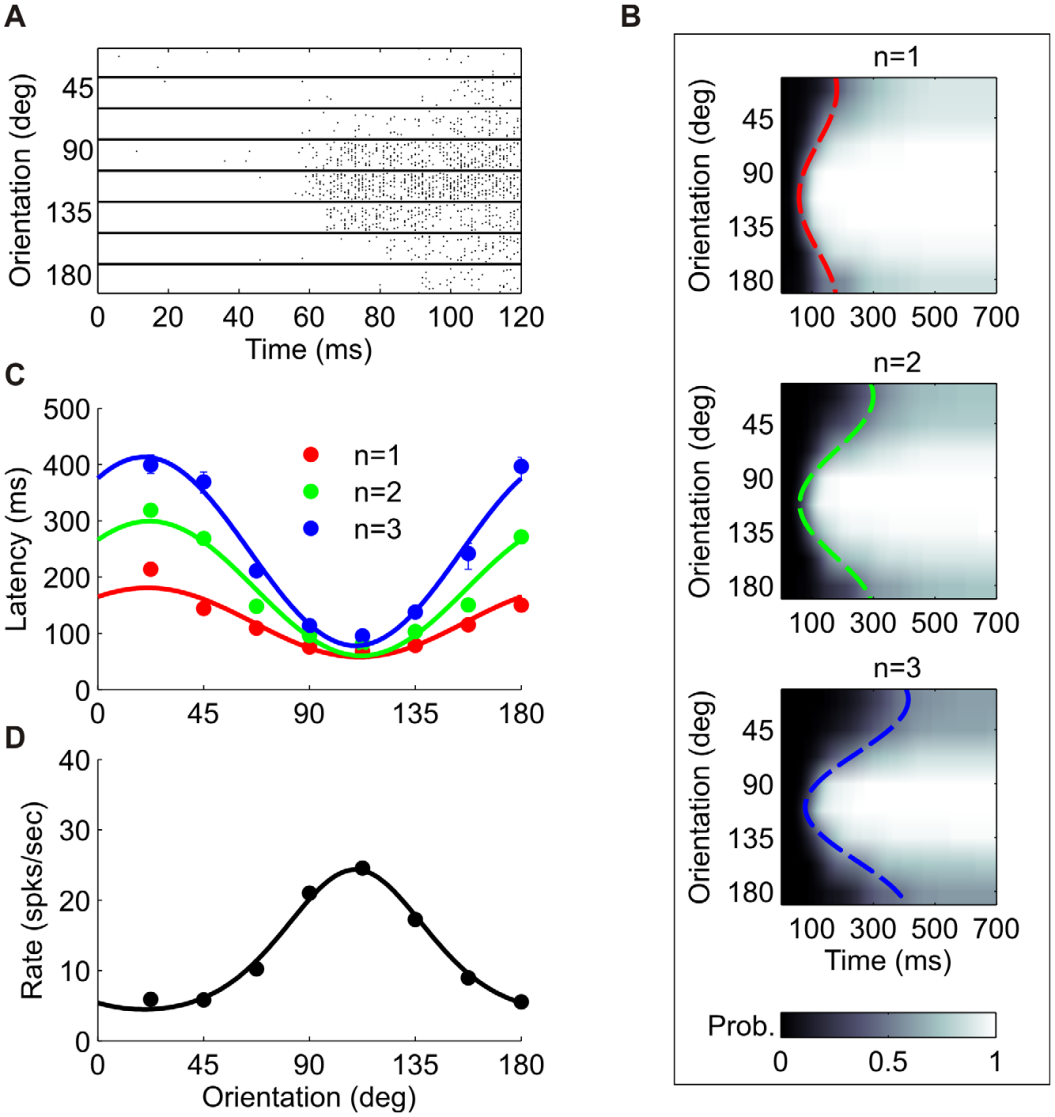
Orientation tuning of spike latencies. (A) Raster plot for of a sample cell in the data (taken from dataset 1 in Table 1). For each orientation, 100 randomly chosen trials (out of 400) are shown. For clarity, only the first 120 ms after stimulus onset are shown. Stimulus duration was 400 ms. (B) Cumulative distribution functions of first, second and third spike latencies (*n* denotes the spike number) for the same neuron. Each row corresponds to a different stimulus orientation and the gray levels represent the probability of the spike occurring before the time indicated on the abscissa. (C) Tuning curves of first, second and third spike latencies, computed as level curves of the corresponding cumulative distributions at 0.5. Cosine fits are shown as solid lines and are also shown as dashed lines in (A). (Error bars were calculated according to the method described in Materials and Methods, but are often smaller than the marker size). (D) Rate tuning curve for the same cell over the entire stimulus duration (black circles) and a fitted von-Mises function (solid line). (Error bars were calculated using the standard error of the mean, but are smaller than the marker size).

We first investigated the tuning of first spike times to stimulus orientation. Figure 1A presents eight raster plots showing the response of the same V1 neuron to eight different orientations of the visual stimulus. Qualitatively, both response strength and response latency seem tuned to the stimulus. Measuring latency by simply calculating the mean time to the first spike is problematic because stimuli that evoke weak responses may result in no spikes on some trials. A more principled approach is to incorporate both response time and probability of firing by computing the probability density function and the corresponding cumulative distribution function of the first spike latency.

Figure 1B (upper panel) shows the cumulative distribution function, 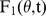; i.e., the probability of firing the first spike before time 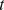 for a given orientation 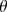 (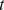 is measured with respect to the onset of the external stimulus). It is convenient to think of the level curves of this function, 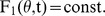, as tuning curves of the neuron. For instance, Figure 1C shows the 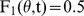 level curve (red circles, fits shown by the solid red line and the dashed line in Figure 1B), which indicates the time at which there was a 50% chance that the neuron had fired its first spike, for each orientation. Typically, the level curves have unimodal orientation tuning, with a single minimum which we define as the latency-based preferred orientation of the cell. Note that although the choice of the 0.5 level curve is arbitrary, similar results were obtained for other criteria. For comparison, the conventional rate-tuning curve of the same neuron is shown in Figure 1D (black circles represent mean firing rates over the entire response, solid curve represents fitted von-Mises function, stimulus duration was 400 ms; see Materials and Methods). The rate tuning is also characterized by a unimodal curve that peaks at the rate-based preferred orientation.

**Figure 2:**
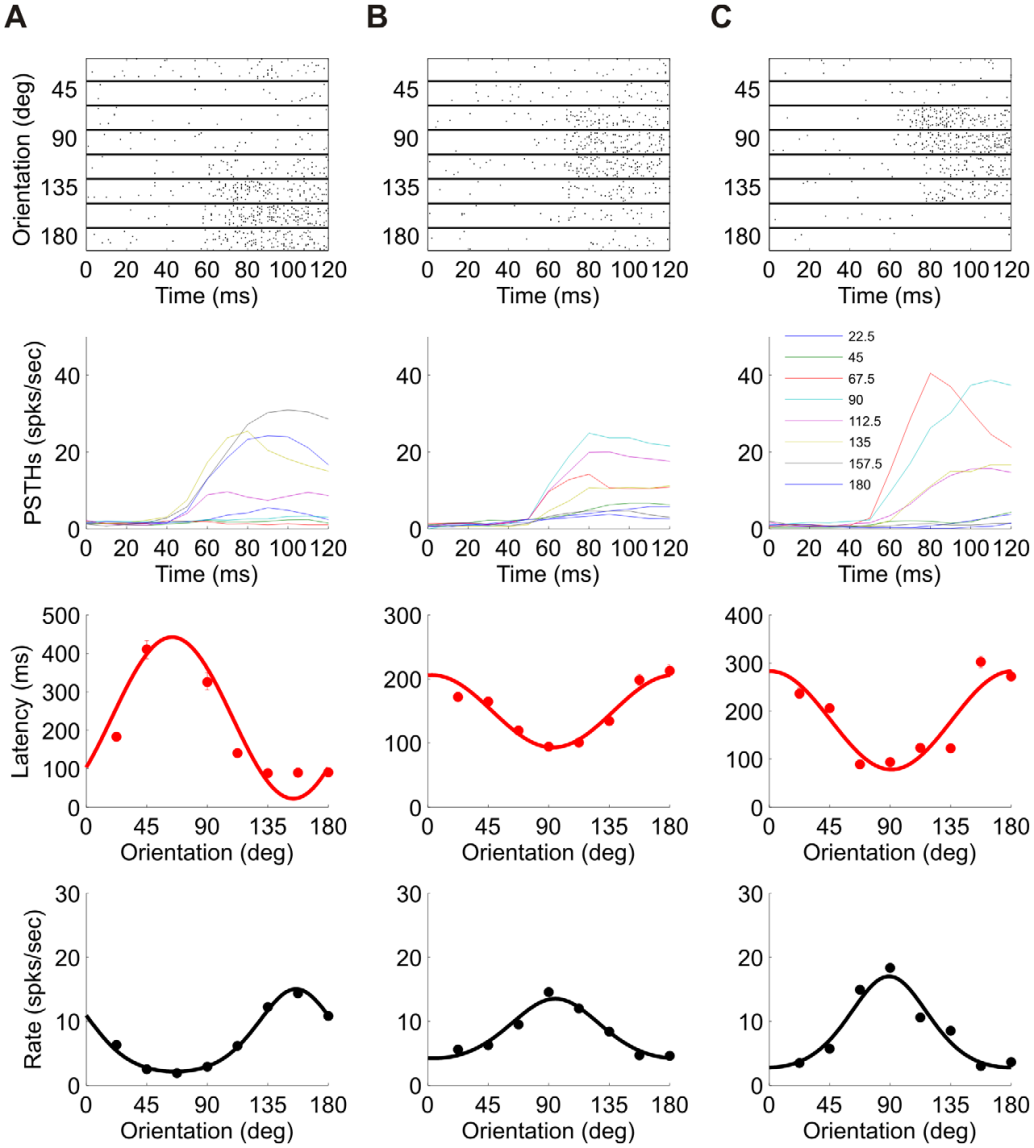
Additional examples of spike latency tuning. Each column, (A)–(C), corresponds to data from a different unit. First row: Raster plot for each of the 8 orientations. For each orientation, 100 randomly chosen trials are shown for 120 ms after stimulus onset. Second row: PSTH (Post Stimulus Time Histogram) for the same time window. Third row: Tuning curve of first spike latency. Cosine fit is shown as a solid line. Fourth row: Rate tuning curve (black circles) and a fitted von-Mises function (solid line). The cell in (A) is taken from dataset 1 in Table 1, in which stimulus duration was 400 ms and the number of trials was 400. The cells in (B) and (C) are taken from dataset 5 in Table 1, in which stimulus duration was 300 ms and the number of trials was 300. These are the same 3 cells as in Figure 5.

Figure 2 shows three additional examples of V1 responses in each column. Eight raster plots for eight orientations are depicted at the top row for each cell. The stimulus dependence of the temporal structure of neural response can be seen from the PSTHs at the second row. The latency tuning curve, in terms of 0.5 level curve of first spike time cumulative distribution, is shown on the third row, and the conventional rate tuning curve appears on the fourth row for comparison. Examining the PSTHs of each cell, one can see that response strength has a considerable contribution to first spike latency, in our definition. For example, in cell B it is mainly the firing rate that is tuned to stimulus orientation. Nevertheless, due to the high firing rate near the preferred orientation, the first spike times tend to be shorter near that orientation. It is also evident that the temporal structure of the response is tuned to the stimulus as well. The modulation of the entire temporal structure (and not a simple temporal shift) limits the ability to extract the ‘pure’ latency tuning. However, as mentioned above, it is the distribution of the *n*th spike time that governs the tWTA readout accuracy; hence, the definition of spike latency used here.

**Figure 3:**
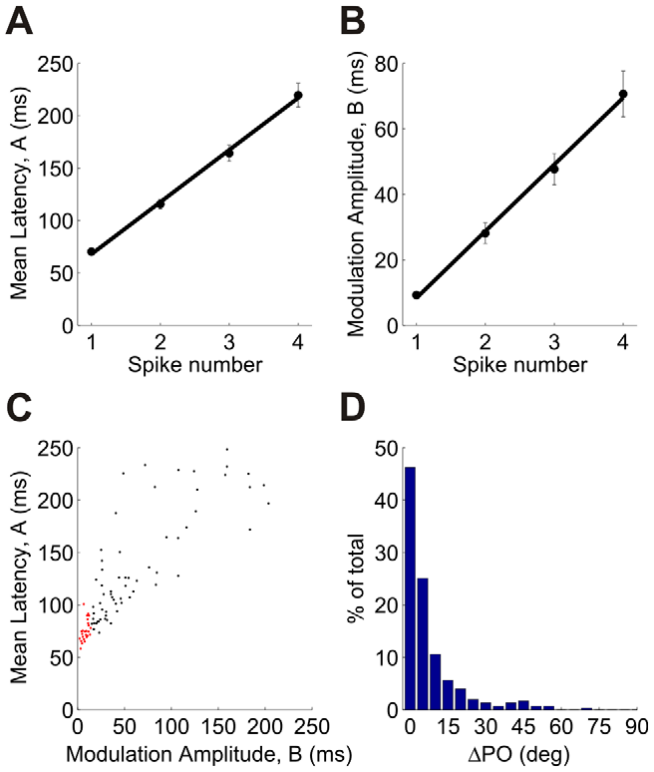
Population statistics of latency tuning. The tuning curves were fitted using a cosine function, 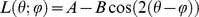, where *θ* is the stimulus orientation and *ϕ* is the latency preferred orientation. (A) Dependence of the mean DC component, *A*, on spike number (averaged over the population). (B) Dependence of the modulation amplitude, *B*, on spike number (averaged over the population). Error bars in (A) and (B) represent ±onestandard error of the mean. (C) A scatter plot of *A* vs. *B* for first spike latency (each point represents one unit; correlation coefficient 0.85). The cells that are marked in red are onset detectors (see text and Figure 4). The statistical analyses in panels (A)–(C) were performed using dataset 3 in Table 1 (159 cells). Similar results were obtained for the other 4 datasets. The correlation coefficients between *A* and *B* were, in decreasing order: 0.88, 0.84, 0.76 and 0.66 (D) Histogram of the difference between the first spike latency-based preferred orientation and the conventional rate-based preferred orientation. In order to avoid artifacts from poorly tuned cells, the histogram shows only cells for which the modulation, *B*, of the first spike latency tuning curve was larger than 15 ms (∼50% of the cells from datasets 1 to 5 in Table 1).

The middle and bottom panels of Figure 1B depict the cumulative distribution function for the second and third spike times, respectively; the green and blue traces in Figure 1C show the corresponding latency tuning curves (level curves at 0.5). The level curve for the cumulative distribution of the *n*th spike time indicates a tradeoff: the curves are delayed in time as *n* increases, but tuning becomes more pronounced. To quantify this behavior we characterized each tuning curve by a ‘DC’ component, denoted by *A*, which represents the mean latency across all orientations, and by the ‘modulation amplitude’, denoted by *B* (see Materials and Methods). Figures 3A and B show the dependence of the mean (*A*) and the modulation amplitude (*B*) of the spike-time tuning curve as a function of *n*, averaged across the population (dataset 3 in Table 1). The delay is evident from the linear increase of *A* with the spike number, while the increase of tuning amplitude, *B*, indicates that the tuning becomes more pronounced as *n* increases. A scatter plot showing the mean latency of the first spike against the tuning modulation of the first spike indicates that they are correlated (Figure 3C; correlation coefficient 0.85). This is a manifestation of an empirical result that the first spike latency at the preferred orientation (*A*–*B*) is approximately constant, and thus neurons with larger modulation amplitudes also have larger mean latencies. Note, that because (*A–B*) is the fitted latency at the preferred orientation and is expected to be positive, we would expect that in general *A* will be larger than *B*. We find that, typically, the rate-based preferred orientation is very close to the latency-based preferred orientation. Figure 3D shows the distribution of the difference (in absolute value) between the rate and the latency preferred orientations of cells with a tuned first spike latency. In about 90% of the cells this difference is less than 20°.

In summary, the latency to the first spike is stimulus dependent: it is shortest for the same orientation that evokes the highest firing rate in the cell. Defining response latency by the first two or three spikes, rather than the first single spike, results in tuning with the same preference but with deeper modulation. Thus, spike latency appears to contain useful information about stimulus orientation.

### Generating a reference signal to measure spike latencies

**Figure 4:**
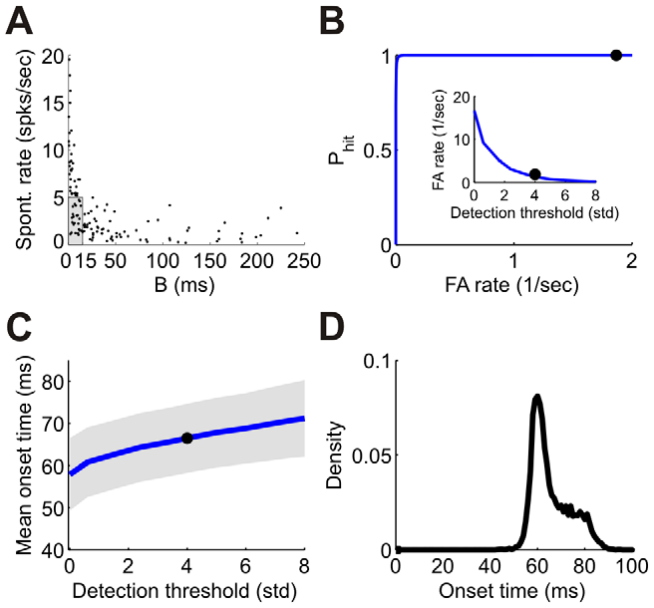
Onset detection. (A) Mean spontaneous firing rate vs. the modulation amplitude, *B*, of the first spike latency tuning curves. Each point corresponds to a single neuron. All neurons in this figure were taken from the same dataset (dataset 3 in Table 1). Neurons were categorized as onset detectors if the modulation was smaller than 15 ms and the spontaneous rate was below 5 spks/sec (shaded box in the lower left corner, 25 cells). (B) ‘ROC’ curve of the onset detection mechanism for a time window of 20 ms. The inset shows the false alarm rate as a function of detection threshold in standard deviations. (C) Mean onset time as a function of detection threshold. The gray band represents ±1 standard deviation. The black circles in (B) and (C) mark the detection threshold of 4 standard deviations above baseline, which we use throughout the paper. (D) Distribution of onset times. The number of spikes was required to be 4 standard deviations above the mean number of spikes in a 20 ms window during spontaneous firing.

Because the brain does not have direct access to information about when a stimulus was presented, a reference signal is required to extract information about stimulus orientation from the first spike latency. Such a reference signal can be reported by neurons which are sensitive to the mere onset of the stimulus. An ideal onset neuron is expected not only to have a uniform spike time latency for all orientations, but also a low spontaneous firing rate, to prevent false alarms. In fact, several neurons in the data showed weak orientation tuning of their first spike latency as well as a low spontaneous firing rate. Figure 4A shows a scatter plot of the spontaneous firing rate against the modulation amplitude, *B*, of the latency tuning curve for a single dataset (dataset 3 in Table 1). We categorized neurons as onset detectors if their modulation amplitude was less than 15 ms and their spontaneous firing rate was less than 5 spks/sec (gray shading in Figure 4A). Typically, we had 10–25 onset detectors in a dataset (10–25% of the population [35]; see Table 1). Because the parameters *A* and *B* are correlated, these neurons also tend to have an earlier latency (Figure 3C, red dots).

In a given trial, onset time was determined using a simple coincidence detection mechanism. Stimulus presence was detected if the group of onset cells fired at least *m* spikes during a time interval of *T* ms, and stimulus *onset* was estimated by the first crossing time of this threshold. A high value of the threshold *m* results in a very low false-alarm rate but compromises the probability of hit, whereas a low value of *m* increases the hit probability but also the false-alarm rate. By varying the *m* criterion we can quantify the Receiver-Operating Characteristic (ROC) curve of this onset detection mechanism; i.e., the dependence of the hit probability on the false alarm rate (Figure 4B). Note that, in contrast to standard two alternative forced choice tasks, in a detection task there are no well-defined trials of ‘no stimulus’, and the stimulus may be absent over a wide range of time intervals. The mean number of false alarms will scale linearly with the duration in which they are counted. Hence, in a detection task, false alarm is measured in rate of occurrence and not in probability. Unless otherwise stated, throughout this paper we use the following parameters for onset detection: a time window of *T* = 20 ms, with a criterion of 4 standard deviations above the mean number of spikes in this time interval during spontaneous firing. This choice takes into account the need for a fast detection of the onset (Figure 4C) while maintaining a high hit probability and a low false-alarm rate. The distribution of estimated onset times (relative to stimulus onset) with this criterion is depicted in Figure 4D. Because the detection of stimulus onset involves a simple integration of spikes emitted by onset detectors, it can be realized in a straightforward way in an integrate-and-fire neuron, producing a similar distribution of onset times (Figure S1).

### The temporal Winner-Take-All Readout

We have shown that first spike latency contains information about stimulus orientation and that there is a distinct subset of neurons whose responses can be used as a timing reference signal. To read out the information embedded in the neural response latencies, we used a temporal Winner-Take-All (tWTA) mechanism, with respect to the above onset mechanism [34]. The complete definition of the method used to compute tWTA performance is provided in Materials and Methods.

The performance of the tWTA is affected by the spontaneous firing rates of the neurons, since the mechanism can erroneously identify a spontaneous spike as an informative one. This effect is reduced by taking a more general readout, the *n*-tWTA, in which the identity of the stimulus is determined by the cell or group of cells that fired the first *n* spikes with respect to the reference signal. This may come at the expense of the time it takes to make a decision. However, if the number of spikes, *n*, is less than or equal to the group size, *N*, then the mean decision time of the *n*-tWTA will be less than the mean first spike time of a single cell, keeping the mechanism fast.

### Discrimination accuracy based on single cell responses

As a first test of the tWTA accuracy we quantified how well it can discriminate between two orientations based on single cell responses. We consider the case where one of the orientations is the cell's preferred orientation θ_0_ (as defined by its latency tuning curve) and the other orientation is θ_0_+Δθ. The tWTA decision rule is to associate the shorter latency with the stimulus at the preferred orientation of the cell and the longer latency with the other stimulus. The probability of correct discrimination, P_C_, using the *n*-tWTA was calculated from the probability density function, f*_n_*(θ,t), of the *n*'th spike latency, as estimated from the data with time relative to the external stimulus onset (see Materials and Methods). Similar to psychometric curves in psychophysical experiments, the curve that describes the probability of a correct response as a function of the orientation difference Δθ is termed the *neurometric curve* of the cell.

**Figure 5:**
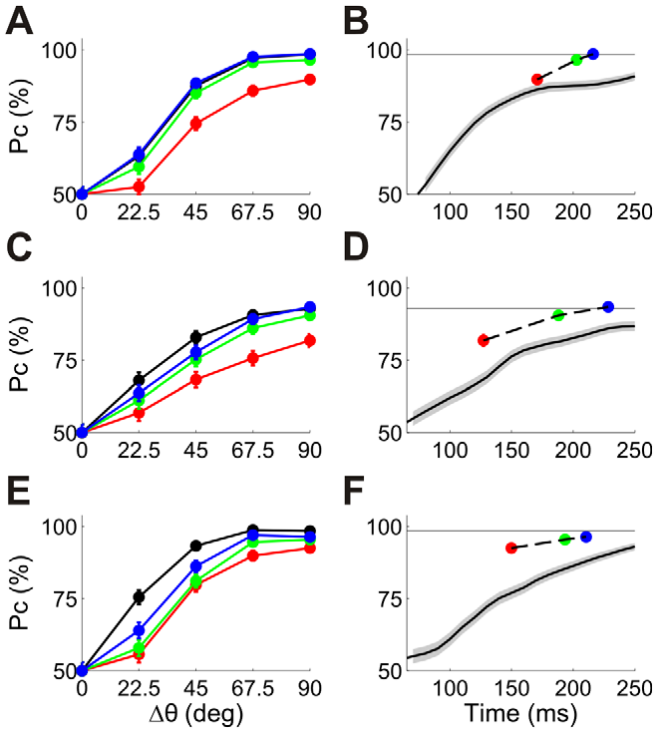
Orientation discrimination using single cell spike latencies and firing rates. (A) Neurometric curves for a single cell using the first spike latency (red), second spike latency (green), third spike latency (blue) and the firing rate (black). These curves represent the probability of correct discrimination in a 2AFC paradigm where one stimulus is at the cell's preferred orientation, PO, and the other at PO±Δθ. (Error bars represent the standard error of the mean, but are often smaller than the marker size). (B) Neurometric curves for 90° discrimination as a function of decision time. The black curve represents probability of correct discrimination based on firing rate for different time windows starting at stimulus onset (the curve starts at 60 ms because deviation from spontaneous activity starts at about this time). (The gray band represents ± standard error of the mean). The horizontal line represents the asymptotic performance using firing rate from the full response (black circle at 90° in A). The filled circles represent decisions using first, second and third spike latencies with the same color code as in (A) (error bars are smaller than the marker size). Each circle is plotted at the corresponding mean decision time. This cell was taken from dataset 1 in Table 1. (C)–(F) The same as (A) and (B) for two other cells from dataset 5 in Table 1. (The 3 cells in this figure are the same cells as in Figure 2).

**Figure 6:**
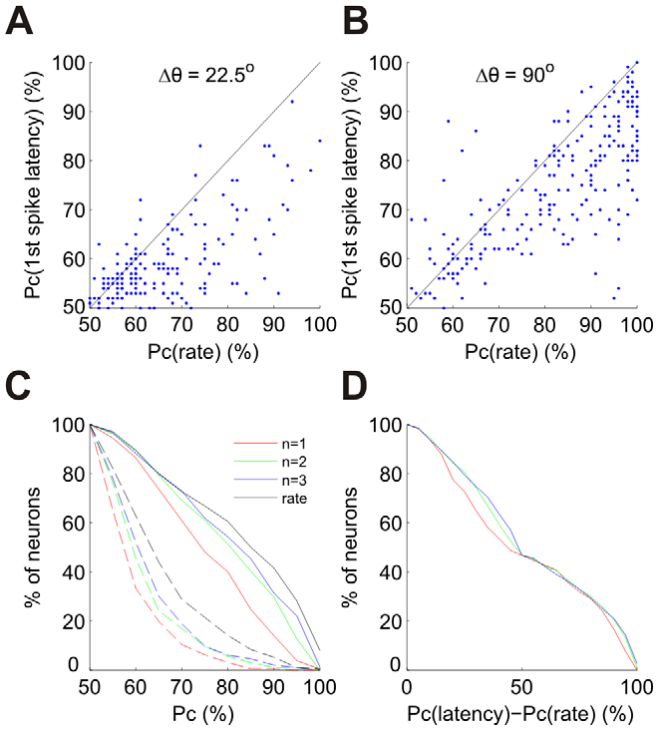
Statistics of orientation discrimination using single cell spike latencies and firing rates. (A)–(B) Pc (probability of correct discrimination) using first spike latency vs. Pc using the spike count of the entire response. Each point corresponds to a single cell. The identity line is shown for comparison (solid black line). (A) Comparison of performance at a fine resolution discrimination task, Δθ = 22.5°. (B) Comparison of performance at a coarse resolution discrimination task, Δθ = 90°. (C) Proportion of cells above a given performance level. The dashed curves correspond to a 22.5° discrimination task and the solid curves to a 90° discrimination task. Different curves correspond to first spike latency (red), second spike latency (green), third spike latency (blue) and firing rate from the entire response (black). (D) Comparison of latency and rate performance at a given decision time. The abscissa is the difference between Pc using the *n*'th spike latency and Pc of the conventional rate code readout, where the rate is estimated from the spike count in the time window from stimulus onset to the mean decision time using the *n*'th spike latency. These differences correspond to the vertical distances between the circles and the solid black curve in the right panels of Figure 5. The curves show the proportion of cells above a given difference. The color code is the same as in (C). The data for all panels are from the tuned cells (*B*>15 ms) in datasets 1, 2, 4 and 5 in Table 1 (244 cells).

Figures 5A, C and E show the neurometric curves of 3 single cells. The red, green and blue curves correspond to the *n*-tWTA readout for *n* = 1, 2, and 3, respectively. For comparison, we show the neurometric curve of the conventional rate code readout in black (the firing rate was estimated from the total number of spikes fired by the cell during the entire response). Typically, as *n* increases, the performance improves and approaches that of the rate code. Figures 6A and B compare the accuracy of the first spike latency code, in terms of probability of correct discrimination, and the rate code, for a relatively fine discrimination task (Figure 6A; 22.5 deg) and for a coarse one (Figure 6B; 90 deg). Latency and rate code accuracy are correlated and, for the coarse discrimination task, the latency code performance is often comparable to that of the rate code. The cumulative distributions of the accuracy of the different codes in these two tasks are shown in Figure 6C.

Figures 5B, D and F show the accuracy of the rate code as a function of the time used for the discrimination for three example cells (same cells as in Figure 5A, C and E). For comparison we plot the accuracy of the *n*'th spike latency code readout at its mean decision time (see Materials and Methods). On brief timescales, the latency code readout is superior to that of the conventional rate code. To quantify this effect, we show in Figure 6D the cumulative distribution of the difference between the accuracy of the *n*-tWTA and the accuracy of the rate code, as computed at the mean decision time using the *n*'th spike latency. As is clear from the figure, this difference is always positive, emphasizing the superiority of the latency code on brief timescales.

The responses we measured were evoked by drifting gratings. We also recorded and analyzed additional data using flashed static gratings of brief (50 ms) and long (300 ms) durations. These data provided qualitatively similar results (see Figure S2).

### Discrimination accuracy based on population responses

Decisions in the central nervous system are expected to involve large numbers of cells. In large populations, the *n*-tWTA discrimination in a two alternative forced choice paradigm can be thought of as a competition between two ‘columns’ towards a threshold of firing *n* spikes. To study the dependence of *n*-tWTA accuracy on the population size we divided the tuned neurons (*B*>15 ms) into artificial columns of equal orientation width according to the latency-based preferred orientation of the cells (see Materials and Methods). For each pair of columns, we measured the probability of correct discrimination as a function of the number of cells in the population (see Materials and Methods). Importantly, unless stated otherwise, the spike latencies in each trial were measured with respect to the onset detection mechanism described above. Thus, the analysis uses only information that is present in the brain, and, in principle, can be performed by an appropriate neuronal mechanism (see Discussion).

**Figure 7:**
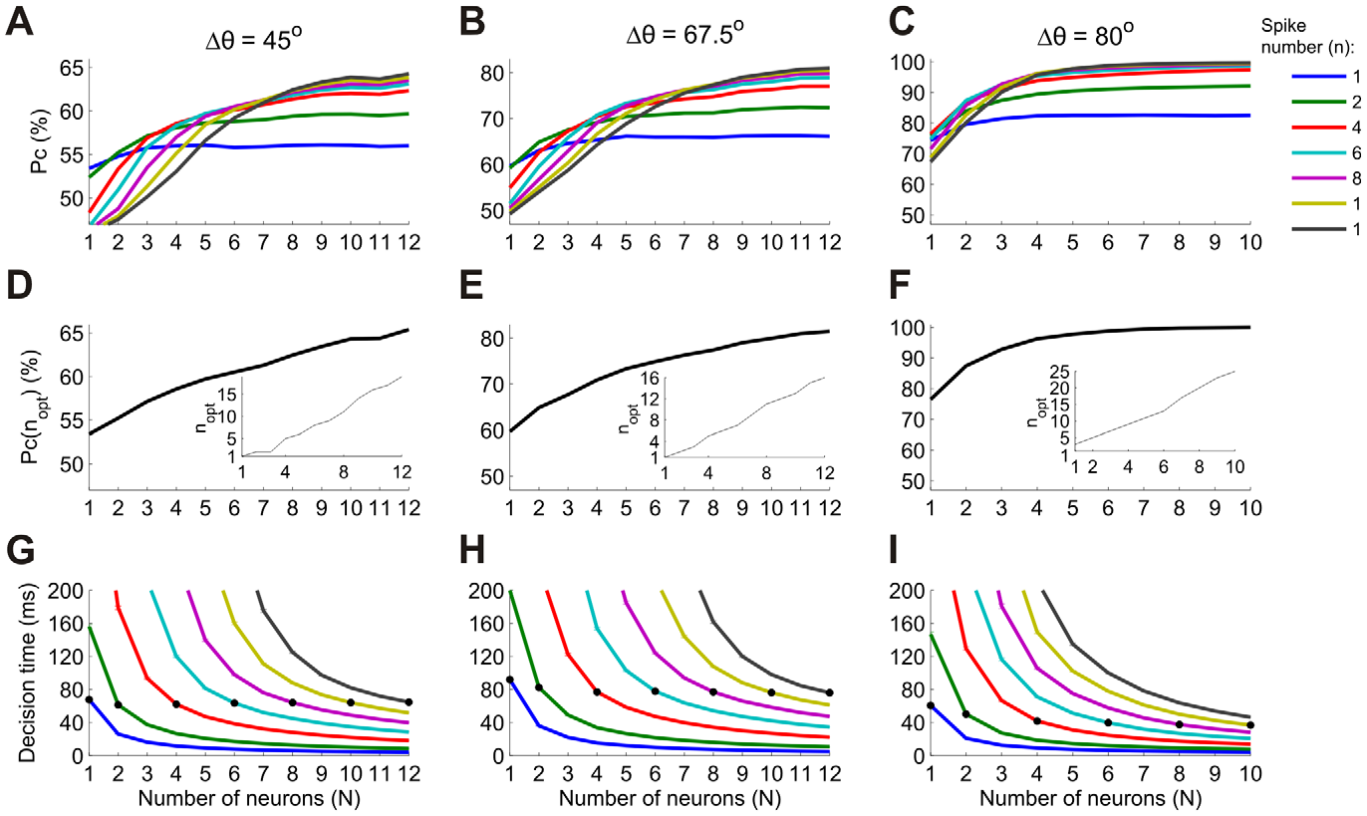
Orientation discrimination using the *n*-tWTA readout in populations of neurons. (A–C) Probability of correct discrimination (Pc) as a function of population size (*N*) for two populations that differ in preferred orientation by 45° (A), 67.5° (B) and 80° (C). Different curves correspond to different values of *n* (see legend). (D–F) Probability of correct discrimination using the optimal value of *n* for each *N* (for the above pairs of populations). The inset shows the optimal *n* for each *N*. (G–I) Mean decision times relative to the onset signal for the neurometric curves in the top panels. (Decision times larger than 200 ms are not shown. Error bars represent ± standard error of the mean). The black circles mark the decision times when *n* = *N*; i.e., when the number of spikes used for the decision is equal to the group size. Note that the data for the left two columns are from dataset 5 in Table 1 whereas the data for the right column are from dataset 3. These datasets had different levels of spontaneous and evoked firing, which are responsible for the differences in the optimal *n* and in the decision times.

Figures 7A, B and C show the *n*-tWTA probability of correct discrimination for three representative pairs of columns as a function of the number of cells in each column, *N*. The pairs of columns differ in terms of the difference between the preferred orientations, 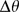. The blue curve depicts the performance of the naïve tWTA (*n* = 1) readout. Initially, for small *N*, tWTA performance increases with *N*. However, beyond a critical size of 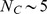, tWTA performance saturates. Theory has shown that two factors may limit tWTA performance. The first is correlations in the first spike latencies of different cells and the second is the spontaneous firing of the cells [34]. We find that although first spike latency is correlated (Figure S3), its effect on tWTA accuracy is negligible (Figure S4; Text S1). The dominant factor that limits accumulation of information from large populations is the spontaneous firing. Clearly, adding more cells also results in adding more spontaneous spikes which interfere with informative spikes (see [34] for a detailed analysis). This effect can be reduced by increasing the decision threshold criterion; i.e., by increasing *n*.

We next analyzed the performance of the *n*-tWTA readout, which takes the winning group to be the first to fire *n* spikes. Different curves in Figure 7A, B and C correspond to different values of the decision threshold, *n*, in the *n*-tWTA readout. In this regime, as *n* is increased the maximal performance is also increased. Figures 7D, E, and F show the performance of the best *n*-tWTA for each *N* (that is, the value of the uppermost curve in a vertical cross-section above this *N*). The inset shows the corresponding value of *n*, 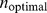, as a function of the population size *N*. As the population size, *N*, grows, it pays to consider more spikes in the readout. Moreover, for these values of population size we obtain that 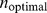 is approximately linear in *N*.

Figures 7G, H, and I show the mean decision time of the *n*-tWTA readout, relative to the onset signal (decision times higher than 200 ms are truncated). As expected, for a given decision threshold, *n*, increasing the number of neurons reduces the decision time significantly. The important point is that the average waiting time for the *n*th spike in a population of *N∼n* cells is around the average waiting time for the first spike of a single cell (black filled circles), which is typically in the range of 40–80 ms. Thus, considering both more spikes and more neurons (*N∼n*) can substantially improve reliability without compromising the decision time.

**Figure 8:**
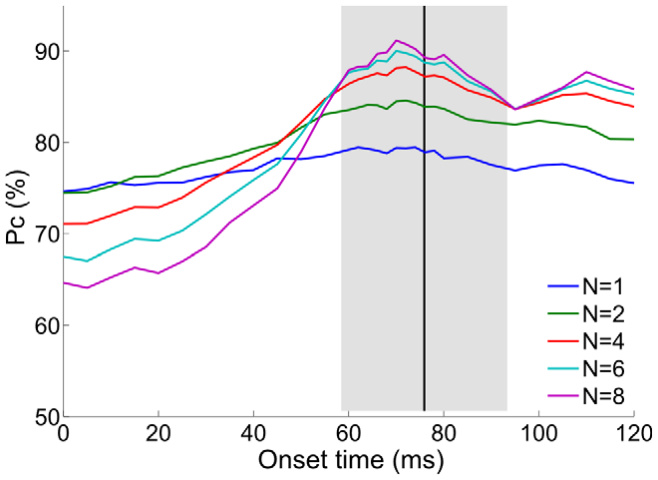
Effect of onset time on orientation discrimination using the first spike latency. To investigate the effect of onset time, we measured the spike times relative to an artificial reference signal. The curves show the probability of correct discrimination (Pc) as a function of onset time for two populations that differ in preferred orientation by 90°. Each curve corresponds to a different population size, *N* (see legend). The black vertical line and the gray band represent the mean onset time ±1 standard deviation using the onset detection mechanism.

In the preceding analysis we measured response timing relative to an internal stimulus onset detection mechanism. We wondered whether performance could be improved by making use of the absolute timing of stimulus onset. In principle, this could decrease the detrimental effect of spontaneous firing [34]. To evaluate this we used an artificial reference signal (i.e. not based on neural responses) which varied from 0 to 120 ms relative to the external stimulus onset. Spike times were then measured relative to this reference signal (spikes before the signal were ignored). Figure 8 shows the accuracy of the tWTA readout (*n* = 1) as a function of the onset time. Estimating the onset too early causes the readout mechanism to consider more spontaneous spikes which only contribute noise. Overestimating the onset time results in a loss of informative spikes. The performance is thus non-monotonic. Since most cells start responding about 60 to 90 ms following stimulus onset, tWTA accuracy peaks at about this time, at a performance level comparable to that achieved using the internal onset detection signal. For comparison, Figure 8 also shows the mean time (±1 standard deviation) of our onset detection mechanism for the same dataset. As can be seen, the onset detection mechanism matches the range of times that produce optimal performance. We conclude that the speed and accuracy of our decoding is similar to that which would be achieved by making use of absolute information as to when the stimulus was presented.

### Discriminating multiple alternatives

**Figure 9:**
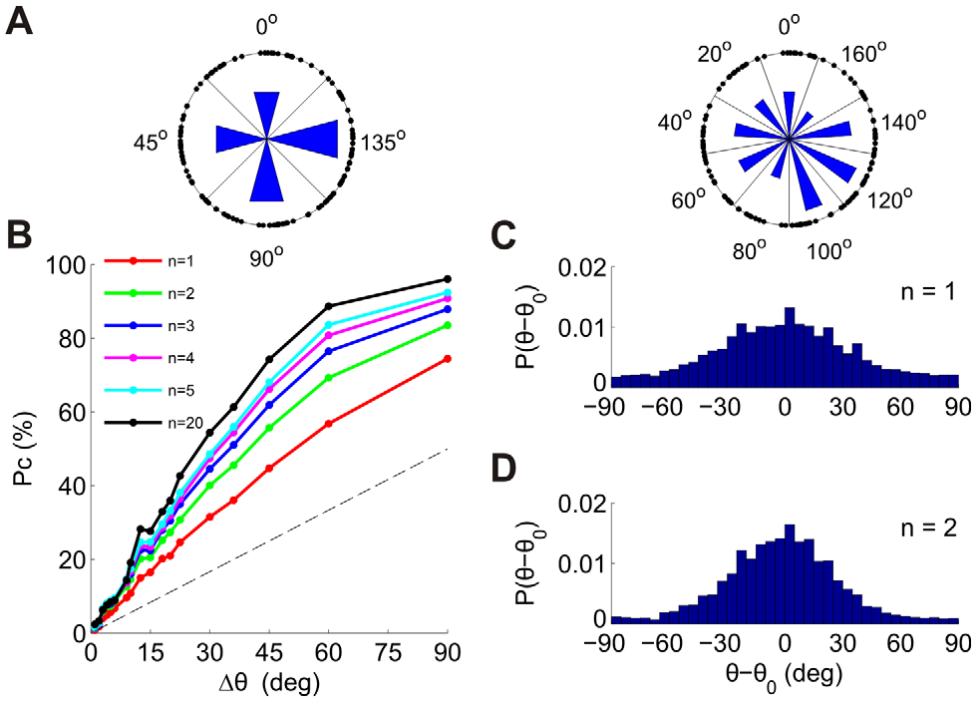
Discrimination among multiple alternatives using the *n*-tWTA in populations of neurons. (A) The tuned neurons in one of the datasets (dataset 3 in Table 1) were divided according to their preferred orientations into *M* groups of equal orientation width, Δθ = 180°/*M*. To illustrate this division, each point on the circle represents a neuron (the angle is twice the preferred orientation). The left plot illustrates division into *M* = 4 groups of width Δθ = 45° and the right plot illustrates division into *M* = 9 groups of width Δθ = 20°. Each group is labeled by the orientation of its center. The lengths of the blue bars are proportional to the number of neurons in each group. (B) Probability of correct discrimination of the *n*-tWTA as a function of group width. The different curves correspond to *n* = 1,2,3,4,5 and 20. (C–D) Distribution of errors for group width of Δθ = 1° for *n* = 1 (C) and *n* = 2 (D).

We next studied the issue of tWTA accuracy in a multiple (*M*)-alternative-forced-choice task using the following setting. All the tuned neurons (*B*>15 ms) in each dataset were divided into *M* ‘columns’ according to their preferred orientation, as depicted in Figure 9A (see Materials and Methods). Note that the number of cells in different groups is not identical and that dividing them into many groups may result in some that contain no cells. The orientation label of each column was defined as the center of that column. The *n*-tWTA decision in a competition among *M* columns was defined as the orientation label of the first column to reach a threshold of *n* spikes. The resolution of this decision is inversely related to the number of alternatives, 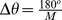. Figure 9b shows the probability of correct discrimination of the *n*-tWTA as a function of 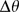 in one of the datasets. Different curves correspond to different values of *n*. The dashed line represents chance value, which is inversely proportional to the number of alternatives. As the decision threshold, *n*, is increased, *n*-tWTA performance improves. This improvement is more significant for coarse discrimination tasks; i.e., for large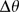.

To gain more insight, Figure 9C depicts the distribution of errors in a fine discrimination task (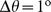) using the tWTA (*n* = 1). The error distribution is very broad and there are relatively many large errors. These large errors are related to spontaneous firing and reflect the fact that discrimination at fine resolutions involves a competition among many groups (180 in this case). In a substantial fraction of the trials the winning group is the first to fire a *spontaneous* spike, which carries no information about the stimulus; hence errors in these cases are distributed uniformly. Using the *n*-tWTA readout with *n* = 2 decreases this effect and makes the distribution narrower, as depicted in Figure 9d. Nevertheless, the decision is still based on a competition between one “correct” group and many (*M*−1 = 179) “incorrect” groups. The chances that one of the “incorrect” groups will fire its first two spikes before the “correct” group are still high and the distribution of errors is still relatively wide. With larger groups of neurons in each bin (i.e. with more samples than that provided by our microelectrode arrays), the decision threshold, *n*, could be increased so as to improve performance for these more difficult discriminations. Nevertheless, for our dataset, we can conclude that *n*-tWTA can perform coarse discriminations remarkably quickly and with high accuracy.

## Discussion

We performed a quantitative analysis of spike latency coding of orientation in primary visual cortex. We found that spike time latency is tuned to the orientation of visual stimuli. Surprisingly, for many neurons, the performance of a WTA decoder based on spike latency was comparable to the performance based on the total spike count during the entire response. This decoding could be performed by measuring latency relative to a reference signal in cortex, namely the pooled responses of a subset of neurons with low spontaneous rates and poor latency-based selectivity for orientation. Performance of the decoder could in principle be improved by using larger populations of neurons. We found that spontaneous firing limits the ability to accumulate information from the spike time latencies of large cell populations, but this can be overcome by scaling the decision threshold linearly with the population size.

### Tuning of spike latencies

Coding of visual attributes by spike latencies was studied previously in the context of contrast processing [26], [31], where it was demonstrated that higher stimulus contrast results in shorter response latency. However, some confusion exists in the literature as to the tuning of first spike latency to the orientation of visual stimuli. Whereas Celebrini et al [32] reported tuning of spike latency of V1 neurons to orientation, Gawne et al. [26] claimed that stimulus orientation mainly modulates response strength and only weakly affects response latency [26].

We have shown that first spike latencies of V1 neurons are tuned to the orientation of external stimuli. This tuning is typically unimodal and the minimal latency is close to the orientation that evokes the maximal firing in the cell. The apparent discrepancy with Gawne et al. is due to different definitions of response latency. In their study, Gawne et al. [26] defined response latency to be the time at which the PSTH reaches half of its peak. The utility of this measure is that it attempts to estimate changes in the ‘pure latency’ in a manner that is unaffected by the changes in the firing rate of the cell. However, since firing rate is modulated by orientation, this definition may measure the latency to a single spike at the null orientation and the latency to ten spikes at the preferred orientation. Hence, using this definition should result in flatter latency tuning curves. Indeed, when applying this definition to our data, we found little modulation of latency with orientation (Figure S5). Moreover, since response strength, the temporal structure of the PSTH, and response latency itself may all be modulated by the stimulus, it is very difficult to obtain a reliable estimate of ‘pure latency’ tuning based on finite amounts of data.

Here we took a more pragmatic approach. Since we are interested in the issue of decoding neural responses on brief time scales, we studied latency tuning using the probability density function of first spike time, which is the quantity that governs tWTA accuracy. Our results thus hold regardless of whether differences in first spike latency arise entirely from differences in response strength, or whether there is some tendency for neurons' absolute latency to vary with stimulus conditions.

### Onset estimation

To extract the information embedded in spike latencies, a reference signal is required. Note that a reference signal is also required for decisions based on spike count in order to determine the start of the counting window. In the general case of latency coding, the onset signal gives a natural reference for measuring latency. However, in our case we do not use the absolute response time, but instead only use relative timing, i.e., who fired first. In this case, an important feature of the onset signal is to filter out spontaneous spikes that are not stimulus dependent and hence carry no information (see Figure 8).

In the case of ‘active sensing’, the intrinsic signal of the motor command [36] can, in principle, serve as the onset signal. However, in the case of ‘passive sensing’; e.g., when a child suddenly jumps in front of your car, the onset signal must be estimated from the responses of sensory neurons. Here we suggested a principle by which stimulus onset is estimated by the group of cells that are not tuned to the information that must be processed rapidly. We showed that a simple summation of the responses of ‘onset’ neurons during short time intervals can provide a reliable reference signal, with sufficient accuracy to allow for accurate identification of stimulus orientation. The onset cells were characterized by weak first spike latency tuning, to limit stimulus dependent bias of the estimated onset time, and low spontaneous firing rates to reduce the false alarm rate. Because the tuning modulation and the mean latency are correlated (Figure 3C), these cells also tend to have an early response. However, even if the onset signal arrives slightly after the tuned neurons started to fire, the performance is only mildly decreased (Figure 8). In terms of the identity of the onset cells, one possibility is that these are inhibitory interneurons, which are known to be responsive but poorly tuned [37], [38]. Since these neurons do not project downstream, this would imply that onset detection is performed locally. A similar approach has been applied in the past for the estimation of the onset of auditory stimuli by Chase & Young [24]. The main differences are twofold. One, Chase & Young used a ‘pseudo population’ signal whereas we use simultaneous recordings of real neural populations. Two, we used the responses of a distinguished subclass of cells with weakly tuned first spike latency for our onset signal, whereas Chase & Young pooled the responses of all the cells.

### A fast and simple readout mechanism in the brain

It remains an open question whether the brain employs a latency-based readout like the tWTA. Nevertheless, the utility of the tWTA in our study has been to enable us to investigate and quantify the information embedded in spike time latency. Let us consider, for example, the case of a two alternative forced choice discrimination task, based on a competition between two neurons. At the time of the first spike the tWTA decision is identical to that of the conventional rate-based readout. The advantage of a latency-based readout is clear when both neurons fired one spike in the counting window. In those cases the latency based readout can extract information from the temporal structure of the response, whereas there is no information in the total spike count. A rate code readout will perform better when more spikes were fired, but this results in a slower readout. A recent study reported that the minimal processing time required for visual perceptual decisions in the monkey is about 30 ms [15]. This brief time scale is on par with the processing time of the latency readout, i.e the mean decision time following the internal onset signal (see e.g. Figure 7I).

To test more directly if a candidate readout mechanism is used by the brain one would need to correlate the behavior of animals with the relevant aspects of neural activity. In a recent study [39], activity of single neurons in monkey V1 was measured together with reaction times for visually guided saccades. It was shown that first spike latency was correlated with behavior whereas firing rate was not, suggesting that spike latency may indeed serve as a source of information for fast decisions in the brain.

### Implementation of the tWTA readout in the brain

**Figure 10:**
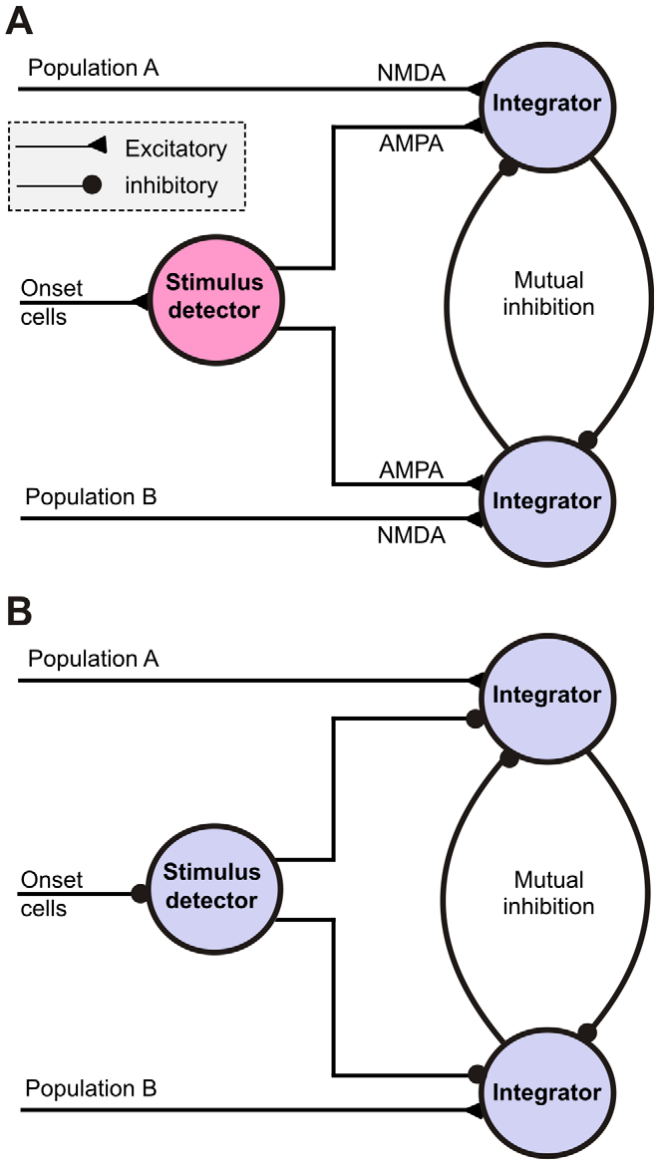
Neuronal architectures for implementing the tWTA readout for a two-alternative task. The figure describes in a qualitative manner neuronal architectures that can implement the tWTA readout in the context of a two-alternative task. The inputs from population A and population B represent the two alternatives. Both architectures rely on reciprocal inhibition for implementing a ‘race to threshold’ competition but they differ in the implementation of the gating mechanism (see Discussion for details). (A) Implementation of the gating mechanism using NMDA synapses. (B) Implementation of the gating mechanism using disinhibition.

As noted above, the implementation of the *n*-tWTA readout requires an integration process and a threshold decision mechanism. In this sense, *n*-tWTA competition is very similar to the ‘race to threshold’ mechanism suggested by Mazurek et al [40], in which the decision in a two alternative task is determined by integrating ‘evidence’ (spikes) for the two competing alternatives to reach a decision threshold (*n* spikes). The decision mechanism involves a winner-take-all type competition, which is an algorithm that others have also used to decode neural response [41]–[43]. Winner-take-all competition can be implemented using reciprocal inhibition between the integrators that represent the different alternatives [44], [45] (Figure 10). Each inhibitory neuron accumulates evidence for the corresponding alternative and fires when it crosses a threshold. Higher threshold values reflect a stricter decision criterion and correspond to higher values of *n* in the *n*-tWTA readout. The integration time constant of the neurons should be on the order of the relevant time scale for decisions (∼10–30 ms).

The circuit also requires a gating mechanism that triggers the integration process based on the reference signal. One qualitative way to implement such a gating mechanism is using NMDA synapses [33] for the tuned inputs (Figure 10A). The inputs from the onset cells are first integrated by a coincidence detector, which in turn excites the inhibitory cells through AMPA synapses (as shown in Figure S1, such a coincidence detector can be implemented using a simple integrate-and-fire neuron). Only when this detector is active, the inhibitory cells become depolarized and the magnesium block of the NMDA synapses is removed, allowing for integration of the tuned inputs. When the onset cells are silent, the NMDA synapses do not allow inputs from the tuned populations to be integrated. The gating mechanism can also be implemented using a disinhibition pathway (Figure 10B). In this case the onset cells are assumed to be inhibitory. Their inputs are integrated by a neuron which inhibits the competing neurons. Thus, the competing neurons are released from inhibition only when the onset cells are active, allowing the ‘race to threshold’ to begin.

Previous studies have proposed more sophisticated mechanisms to combine information from the first spikes of different neurons in a large population. These methods include rank order [30], [46] and synfire chains [47]. The utility of tWTA is that its simplicity enables statistical analysis of its accuracy, whereas sophisticated readout mechanisms that rely on specific combinations of firing orders cannot be tested with finite data on the order of a few hundred repetitions per stimulus condition. Furthermore, these readouts may be more difficult to implement in biological circuits.

Recently first spike latency code has been analyzed in the framework of fast discrimination of sound source location in the auditory system [48]. There are several interesting similarities and differences worth noting. In both systems, many cells exhibit tuning of their first spike latency to the stimulus. Tuned cells are typically characterized by a unimodal latency tuning curve that peaks close to the preferred stimulus of the cell, as defined by the rate tuning curve. In addition, the accuracy of first spike latency readout is typically comparable though somewhat inferior to the accuracy of the conventional rate code in single tuned cells in both systems. The main differences between the systems are the higher spontaneous firing rates in visual cortex and the poorer performance of V1 neurons for orientation discrimination. To overcome the detrimental effect of spontaneous spikes, we developed here a novel onset detection mechanism, based on pooling the responses from a set of simultaneously recorded neurons. The use of simultaneous data from array recordings rather than single units also enabled us to investigate the accuracy of latency coding at the population level without the use of artificial pseudo populations of neurons.

In summary, our study demonstrates that the orientation tuning of first spike latencies enables accurate discrimination of orientations on brief time scales. Spontaneous firing limits the resolution of the decision. However, larger populations can afford better resolution. Furthermore, in many cases when fast decisions are essential, it is important that the probability of correct response will be high but coarse resolution may suffice. This may be a general principle used by the nervous system when fast decisions are essential. For example, when an object suddenly appears on the road while we are driving, all we need to know is its rough location. In most cases we react before we realize whether this object is a child, a dog or just a plastic bag. These finer details can be sorted out later as more spikes are accumulated using readout mechanisms that take into account the entire neural response.

## Materials and Methods

### Ethics statement

All procedures were approved by the Institutional Animal Care and Use Committee at the Albert Einstein College of Medicine of Yeshiva University, and were in compliance with the guideline set forth in the United States Public Health Service *Guide for the Care and Use of Laboratory Animals*.

### Experimental procedures

The methods we use to record from neural populations have been described in detail [49]. In short, we recorded from anesthetized (sufentanil citrate, typically 6–18 microg/kg/hr, adjusted as needed for each animal), paralyzed (vecuronium bromide, 0.1 mg/kg/h) macaque monkeys (macaca fascicularis). Vital signs were monitored continuously to assure adequate anesthesia and the well-being of the animal. The pupils were dilated with topical atropine and the corneas protected with gas-permeable hard contact lenses. Supplementary lenses were used to bring the retinal image into focus.

Neural activity was recorded using the Cyberkinetics “Utah” Array (Cyberkinetics Neurotechnology Systems), using methods reported previously [49],[50]. The array consists of a 10×10 grid of silicon microelectrodes (1 mm in length) spaced 400 µm apart, thus covering 12.96 mm^2^. The array was inserted roughly 0.6 mm into cortex using a pneumatic insertion device [51], resulting in recordings confined mostly to layers 2–3. Signals from each microelectrode were amplified and bandpass filtered (250 Hz to 7.5 kHz). Waveform segments that exceeded a threshold (periodically adjusted using a multiple of the rms noise on each channel) were digitized (30 kHz) and sorted off-line. Sorted units included both well-isolated single units and small multiunit clusters. Neuronal receptive fields were roughly 2–5° from the fovea.

Visual stimuli were displayed at a resolution of 1024×768 pixels and a video frame rate of 100 Hz on a calibrated CRT monitor. Stimuli were oriented drifting gratings presented in a circular aperture surrounded by a gray field of average luminance (8 orientations in 4 datasets and 36 orientations in one dataset). Stimuli were presented binocularly, for 300–400 ms, and separated by 500–800 ms intervals during which we presented an isoluminant gray screen. Stimulus orientation was block randomized, and each stimulus was presented 200–400 times (see Table 1 for details). In 4 datasets the initial phase of the drifting grating was identical across trials. To test whether our results were skewed by this, we collected and analyzed additional data using initial phases that were randomized across trials. We obtained similar results from this dataset (see Figure S6). To verify that our results also generalize to static images, we collected and analyzed responses to static gratings presented for 50 or 300 ms (dataset 6 in Table 1). We obtained similar results from this dataset (see Figure S2).

### Rate tuning

The rate tuning curves represent the mean firing rate across all trials at each orientation. The firing rate in a trial was calculated using a time window from stimulus onset to 300 ms after stimulus offset. The tuning curves are well fitted using the Von-Mises function:
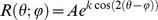
where *θ* is the stimulus orientation and *ϕ* is the rate-preferred orientation of the cell.

### Latency tuning

To generate latency tuning curves for a neuron we first estimate the probability density function of the first spike latency of this neuron, f_1_(θ,t). This is done by computing the histogram of the first spike times over trials and then normalizing it. Note that because in some trials there may be no spikes, the integral of the probability density function may not be 1 but slightly below. The spike times are measured with respect to the external stimulus onset and the histogram is generated using bins of 10 ms from time 0 to 300 ms after stimulus termination. The corresponding cumulative distribution, *F*_1_(θ,t), is generated by direct numerical integration of the density function. A similar procedure is applied to obtain the *n*th spike time probability density, *f*_n_(θ,t), and cumulative distribution, *F*_n_(θ,t), for general *n*.

The latency tuning curve of the *n*'th spike is defined as the level curve at 0.5 of the corresponding cumulative distribution function. These level curves are fitted using a cosine function of the form:
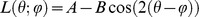
where *θ* is the stimulus orientation and *ϕ* is termed the latency preferred orientation of the cell. Parameter *A* represents the mean latency and *B* represents the modulation of the tuning.

Here, the reference time is chosen to be the onset of the external stimulus, but in principle other external reference times can be used, e.g. 20 ms after stimulus onset. We note that in the cosine fit, changing the reference time will change the value of *A* but not *B*. The arbitrary choice of the reference is also why a simple cosine function is more appropriate here than the von-Mises function. Choosing the reference such that at some orientations the latency is zero requires parameter *k* at the von-Mises function to diverge to infinity. In addition, if the latency is negative with respect to the reference at some orientations, the von-Mises function will not fit at all, as it is purely positive.

Because in some trials there may be no spikes, error bars for the latency tuning curves cannot be simply calculated from the standard error of the mean associated with the spike times. In order to generate error bars, we first calculated the standard errors of the mean for the cumulative distribution, *F*. This can be done by noting that *F* is the mean of a Bernoulli variable and thus its variance is 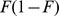. The standard error of the mean is therefore: 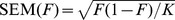, where *K* is the number of trials. We then calculated the level curves at 0.5 for *F*+SEM*(F)* and for *F*-SEM*(F)*, and used them to generate lower and upper error bars, respectively. These error bars are depicted in Figure 1C and in subsequent plots of spike latency tuning.

### Onset detection

In each dataset we identify a group of cells that can serve for the detection of stimulus onset. These cells are characterized by poor tuning and low spontaneous firing rates. The spontaneous firing rates are estimated from the recordings during the inter-stimulus interval (ISI) after each stimulus. From each ISI we remove the first 300 ms, assuming that after this period the cell returned to its spontaneous rate (i.e. any post-response adaptation of spontaneous rate would have dissipated). The tuning is characterized by the modulation amplitude, *B*, of the cosine fit to the first spike latency tuning curve. In each dataset, the cells with a spontaneous rate lower than 5 spks/sec and with a modulation lower than 15 ms, were labeled as onset detectors. Using this definition, the number of onset detectors in a dataset is roughly 10–25% of the population (see Table 1).

The onset signal in each trial is generated using coincidence detection. We used a running time window of *T* ms and looked for the first time in which there were at least *m* spikes in this window (but see also Figure S1). The onset time is then defined as the end of this window. To set *m*, we first estimated the mean and standard deviation of the number of spikes that these cells fire in a time window *T* during spontaneous firing. We then set the threshold *m* to be *N*_v_ standard deviations above this baseline value. By varying *N*_v_ for a given *T* we generated ROC curves for the onset detection process. In subsequent analyses we used *T* = 20 ms and *N*_v_ = 4 standard deviations. This onset signal was used as the reference time *t*_ref_ for measuring spike latencies in the tWTA.

### Discrimination accuracy based on single cell responses

The discrimination accuracy of single cells is computed in the context of a Two-Interval 2-Alternative-Forced-Choice paradigm. We assume that the cell is presented with two stimuli, one at orientation θ_1_ and the other at orientation θ_2_, where θ_1_ is the preferred orientation of the cell. The probability that the tWTA will yield the correct response is the probability that the latency of the response to θ_1_ will be shorter than the latency of the response to θ_2_. To find this probability, we multiply the probability that the neuron first fired at time *t* in response to θ_1_ by the probability that it did not fire before *t* in response to θ_2_, and then we sum over all possible times, *t* (the time is measured with respect to the onset of the external stimulus). Formally, this is given by the following integral:
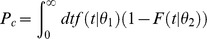
However, recording time is finite. Our data contains only 300–400 ms of stimulus presence and the following 700–800 ms of inter-stimulus time; hence, in some cases the decision threshold is not reached during our recording time. In practice we assume that after time *T*_0_, that contains the stimulus presence time and the initial 300 ms of the following inter stimulus period, the neuron returns to its spontaneous firing rate. Assuming Poisson firing with mean rate λ after time *T*_0_, we obtain:
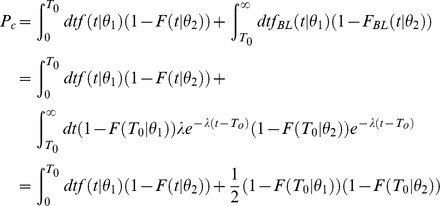
It is also important to note that *f* and *F* are estimated from the data using time bins of Δ*t*. The spikes from the responses to θ_1_ and θ_2_ may fall within the same time bin, leading to correct discrimination at chance level. Correcting for this effect we obtain:
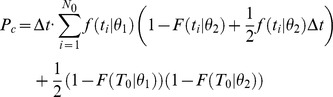
Finally, for general *n*, the correction that stems from the spontaneous firing after response termination is more complicated due to all the combinations of spike trains that have to be taken into account. The general expression is then:
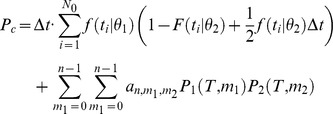
where the coefficients 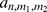 are given by:
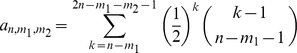
and 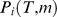 is the probability that neuron *i* fired *m* spikes up to time *T* in response to stimulus θ*_i_*.

The probability of correct response *Pc* is the mean of a Bernoulli variable and the corresponding standard error of the mean can be calculated as 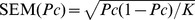, where *K* is the number of trials.

To prevent possible interaction between the discrimination accuracy analysis and the latency tuning analysis, we separated each dataset into a training and test set, each consisting of half of the trials (randomly chosen). The training set was used for estimating the latency preferred orientation of the cell. The test set was then used for constructing the neurometric curve, based on the preferred orientation from the training set.

To calculate the mean decision time we first compute the probability that decision will be made between *t* and *t*+ Δ*t*,
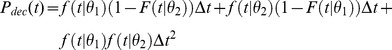
and then compute its mean.

### Discrimination accuracy based on population responses

To study the dependence of *n*-tWTA accuracy on the population size we divided the neurons into several artificial columns of equal orientation width (for datasets with 8 orientations we divided into 8 columns of 22.5° width and for the dataset with 36 orientations (dataset 3 in Table 1) we divided into 9 columns of 20° width). Each neuron was assigned to the column with the closest orientation to its own preferred orientation (the number of neurons in such a column ranged from 1 to 14). For each pair of columns, we then constructed a neurometric curve, which measures the probability of correct response as a function of the number of neurons, *N*.

Given two subsets of *N* cells from each column, we simply went over all trials with the orientation of the first column and then over all trials with the orientation of the second. In each trial, the subset that first fired the *n*'th spike after the onset signal from the onset neurons was the *n*-tWTA. If the time of the *n*'th spike was the same for both subsets we tested whether one of the subsets fired additional spikes in the same bin and took the winner as the subset that had more spikes. The average number of correct responses using the *n*-tWTA gave an estimate of the probability of correct response for these two subsets of cells. For a given *N* we averaged this value over 1000 realizations of the subsets of neurons. The decision time in a given trial was the time relative to the onset signal and we calculated its mean and standard error of the mean across all trials.

### Discrimination among multiple alternatives

To investigate discrimination among multiple alternatives, the neurons were divided according to their preferred orientation into *M* groups of equal orientation width, Δθ. For convenience, we set one group to be centered at the stimulus orientation (e.g., if *M* = 18 and the stimulus orientation is 45°, the centers will be at 5°, 15°, 25°,…, 175°). On a given trial, the group that was first to fire *n* spikes was the *n*-tWTA. If several groups fired the *n*'th spike at the same time we chose among them in a random manner. The error in the trial was the (signed) difference between the orientation of the winning group and the stimulus orientation. The probability of correct response was calculated as the average number of times in which the correct group was the winner.

## Supporting Information

Figure S1 **Onset detection using an integrate-and-fire neuron.** In addition to the onset detection mechanism described in the main text, we investigated an implementation of onset detection in a more biologically plausible architecture, namely by performing the coincidence detection using a leaky integrate-and-fire neuron. The neuron integrates the spikes from the onset neurons with equal synaptic weights until a threshold is reached. For simplicity, the integration process was set such that each spike increases the membrane potential by one unit. The voltage then decays exponentially with a time constant of 20 ms. (A) Illustration of the integration process by a leaky integrate-and-fire neuron. The top trace shows a series of spikes from all onset neurons relative to stimulus onset and the bottom trace show the membrane potential of the integrate-fire-neuron (in arbitrary units) as it integrates these spike. The accumulation of spikes around 45–65 ms after stimulus onset causes the neuron to first cross the specified threshold and an onset is detected. (B) Mean onset time as a function of threshold. The mean is calculated over all trials in one dataset. The gray stripe represents ±one standard deviation. The horizontal line represents the mean onset time using the running window approach which was used in the main text (the time window was 20 ms and the threshold was set to 4 standard deviations above spontaneous firing, which corresponds here to m = 6 spikes). The vertical line represents the required threshold for the integrate-and-fire neuron (3.3) to achieve the same mean onset time. (C) Distribution of the difference in onset times between the two mechanisms across all trials. The threshold for the integrate-and-fire neuron is the one which achieves the same mean onset time as the running window method (3.3).
(TIF)

Figure S2 **Latency tuning and orientation discrimination for static gratings.** (A) First spike latency tuning curves for one cell. Different colors denote different stimulus durations (50 ms and 300 ms; see legend). (B) The corresponding neurometric curves for the same cell. For each stimulus duration, the solid curve corresponds to the first spike latency neurometric curve and the dashed curve to the conventional rate neurometric curve. (C)–(D) Same as (A)–(B) for a different cell. (E) Proportion of cells above a given level of the mean latency, *A*, for the two stimulus durations. (F) Proportion of cells above a given level of the modulation tuning, *B*, for the two stimulus durations. (G)–(H) Statistics of orientation discrimination. (G) Proportion of cells above a given performance level for the 300 ms stimulus. The dashed curves correspond to a 22.5° discrimination task and the solid curves to a 90° discrimination task. Different curves correspond to first spike latency (red), second spike latency (green), third spike latency (blue) and firing rate from the entire response (black). (H) Same as (G) for the 50 ms stimulus. The analyses were performed using dataset 6 in Table 1 (98 tuned cells for the 300 ms stimulus and 89 tuned cells for the 50 ms stimulus).
(TIF)

Figure S3 **Pairwise correlations of response latencies among neurons.** (A) Trial to trial fluctuations of first spike latencies for a pair of cells. For each neuron we calculated a normalized measure of its first spike latency by subtracting the mean latency and dividing by the standard deviation. These cells had similar latency preferred orientations, 139° and 136°, and their normalized first spike latencies are shown for the first 25 trials in which the stimulus orientation was 135°. The first spike latency of the two cells fluctuates from trial to trial around its mean. However, typically, when one cell fires sooner than its mean latency the other does as well, and similarly when the response is delayed, resulting in a positive correlation coefficient of 0.45. (B) Distribution of first spike latency correlation coefficients. For each pair of cells with latency tuning (*B*>15 ms) a correlation coefficient is calculated separately for each stimulus orientation. The mean of this distribution is 0.07 and its standard deviation is 0.04. (C) Dependence of correlations on the difference in preferred orientations (POs). The cell pairs were divided into six groups according to the difference between their preferred orientations, ΔPO. Each point represents the mean correlation coefficient for all pairs of neurons at a given range of ΔPO and the error bars represent the corresponding standard errors. The solid line represents the linear regression and its slope is −10^−4^±2•10^−5^ (deg-1). These results indicate that there is a very weak dependence of the first spike latency correlation on the difference between the latency preferred orientations of the cells.
(TIF)

Figure S4 **Effect of pairwise correlations on tWTA accuracy.** Correlations in the trial to trial fluctuations of spike latencies can affect the utility of pooling information from groups of cells. To quantify the effect of correlations we compared the discrimination performance using the original simultaneous data to the performance using shuffled data with no correlations. In the shuffled data, the responses of different cells on a given trial were taken from different trials in the original data. The onset signal from the onset neurons was also shuffled among trials. For each shuffled version of the data we found the corresponding neurometric curve, and then averaged the result from 50 shuffles. (A–C) Probability of correct discrimination (Pc) as a function of population size (N) for two populations that differ in preferred orientation by 45° (A), 67.5° (B) and 80° (C) (same pairs of populations as in Figure 7). (D) Probability of correct discrimination in a model of two columns (see Text S1). (E–H) The effect of shuffling the responses of neurons in different trials. The curves show the difference in Pc between the original and the shuffled data (Pc(original)-Pc(shuffled)) for each pair of columns on the left. Although the performance is typically better in the original correlated data, the overall size of the effect is relatively small, on the order of 1%. For small populations, the correlations increase tWTA accuracy. However, as the population size increases, this difference decays to zero. The simplified model (H) captures the behavior of the data (see Text S1).
(TIF)

Figure S5 **Effect of different definitions of response latency.** Gawne et al. [26] recorded responses to oriented bars in V1 of behaving monkeys and reported that the effect of stimulus orientation on response latency is relatively weak. The difference with our results can be attributed to different definitions of response latency. Gawne et al. defined response latency to be the time at which the PSTH reaches its half peak (in cases where the peak was less than twice the spontaneous activity, latency was left undefined). We used the cumulative distribution function of first spike times and defined the first spike latency tuning curve as a level curve of this distribution. (A–C) First-spike latency tuning curves as computed by first spike (cumulative) distribution level curves (red) and the corresponding latency tuning curves for the same cells using the time at which the PSTH reaches half of its peak (black). (Lower and upper error bars for the halfmax definition were calculated using the times at which the PSTH reaches half the peak of the PSTH minus and plus its standard error of the mean). *B* denotes the modulation amplitude using our latency definition and *B*′ using the half max definition. For the cells in (A) and (B), the latency tuning curve using the half max definition (black) is relatively flat, whereas the first spike latency tuning curve using our definition (red) shows a strong modulation. For the cell in (C) both definitions show pronounced tuning. (D) Scatter plot of the modulation amplitudes, *B* and *B*′, for each cell. Notably, the tuning amplitudes using the halfmax definition are relatively small, whereas our definition results in many cells with significant modulation.
(TIF)

Figure S6 **Effect of stimulus phase on spike latency tuning.** The panels on the left side (A,C,E,G) depict results from a dataset in which all stimuli of the same orientation had identical initial phases; the panels on the right side (B,D,F,H) depict results from a dataset in which the initial phases were random. The two datasets were recorded in the same animal using the same electrode array. (A–B) PSTHs of two single cells. The stimuli were near the preferred orientation of the cells and the PSTH was constructed from 300 repetitions. For fixed initial phase, the PSTH is characterized by a periodic modulation whereas for random phases there is no such modulation. (C–D) First spike latency tuning curves of the neurons in (A) and (B). (E–F) Distribution of latency tuning modulation amplitude, *B*, in the two datasets. The substantial tuning of the response latency in the random phase dataset cannot be attributed to the stimulus initial phase. The inset in (F) shows the two cumulative distribution functions (black for the fixed phase dataset and blue for the random phase dataset). The similarity of the two distributions indicates that there is no significant difference between the orientation tuning levels of first spike latencies in the two datasets. (G–H) Mean neurometric curves using the first spike latency (red), second spike latency (green), third spike latency (blue) and the firing rate (black). Error bars represent the standard deviation and are shown only for the rate and the first spike latency neurometric curves. The fact that performance is similar indicates that our results reflect the tuning of the first spike latency to stimulus orientation and not the initial phase of the stimulus.
(TIF)

Text S1 **A simple model for studying the effect of pairwise correlations on tWTA accuracy.**
(DOC)
