# Supplementary figures and images for: Fast Coding of Orientation in Primary Visual Cortex
Source: PLoS Comput Biol. 2012 Jun 14;8(6):e1002536. doi: 10.1371/journal.pcbi.1002536 (PMC3375217; doi:10.1371/journal.pcbi.1002536)

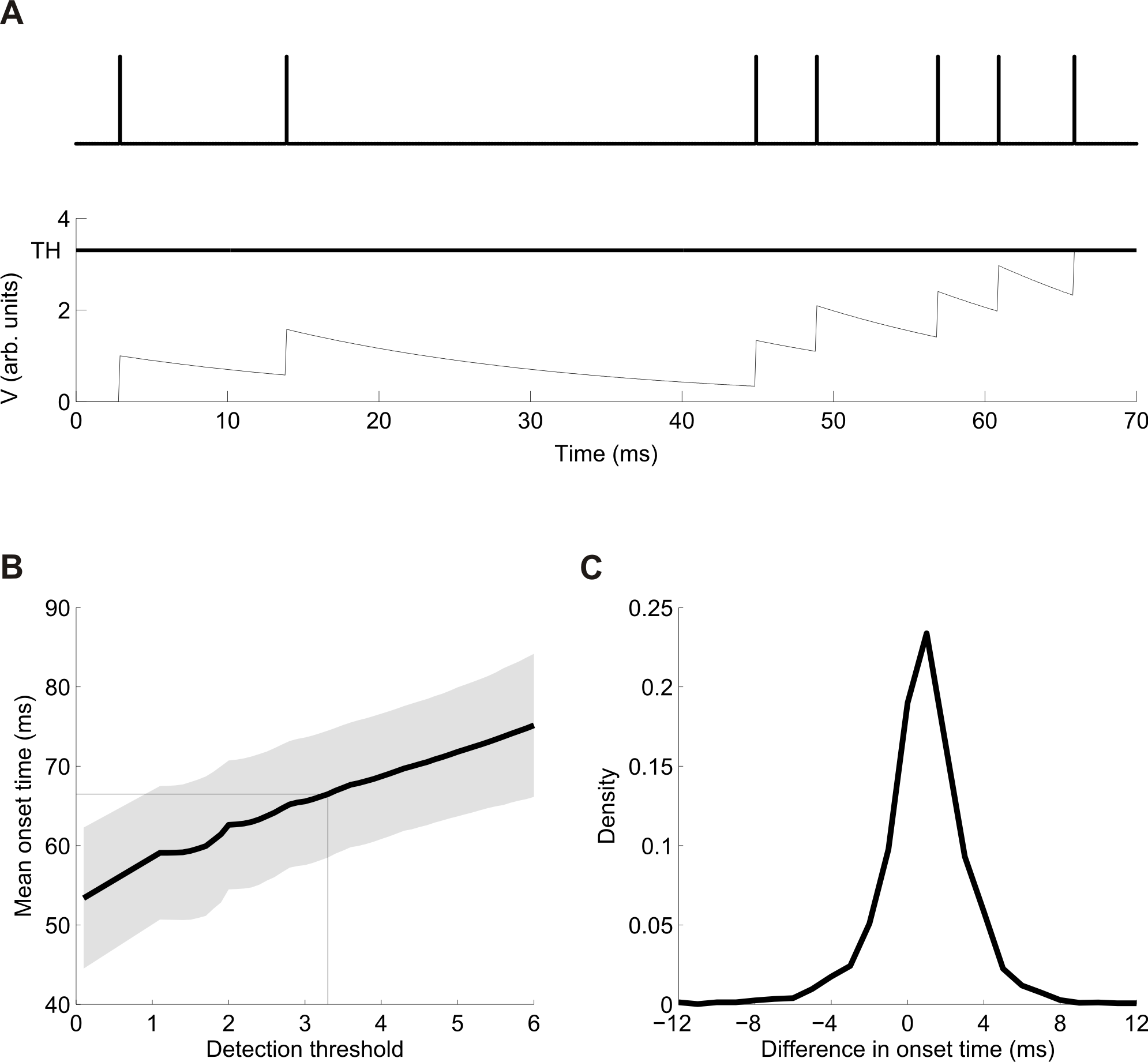

Supplement: Figure S1 — Onset detection using an integrate-and-fire neuron. In addition to the onset detection mechanism described in the main text, we investigated an implementation of onset detection in a more biologically plausible architecture, namely by performing the coincidence detection using a leaky integrate-and-fire neuron. The neuron integrates the spikes from the onset neurons with equal synaptic weights until a threshold is reached. For simplicity, the integration process was set such that each spike increases the membrane potential by one unit. The voltage then decays exponentially with a time constant of 20 ms. (A) Illustration of the integration process by a leaky integrate-and-fire neuron. The top trace shows a series of spikes from all onset neurons relative to stimulus onset and the bottom trace show the membrane potential of the integrate-fire-neuron (in arbitrary units) as it integrates these spike. The accumulation of spikes around 45–65 ms after stimulus onset causes the neuron to first cross the specified threshold and an onset is detected. (B) Mean onset time as a function of threshold. The mean is calculated over all trials in one dataset. The gray stripe represents ±one standard deviation. The horizontal line represents the mean onset time using the running window approach which was used in the main text (the time window was 20 ms and the threshold was set to 4 standard deviations above spontaneous firing, which corresponds here to m = 6 spikes). The vertical line represents the required threshold for the integrate-and-fire neuron (3.3) to achieve the same mean onset time. (C) Distribution of the difference in onset times between the two mechanisms across all trials. The threshold for the integrate-and-fire neuron is the one which achieves the same mean onset time as the running window method (3.3). (TIF) [file pcbi.1002536.s001.tif]

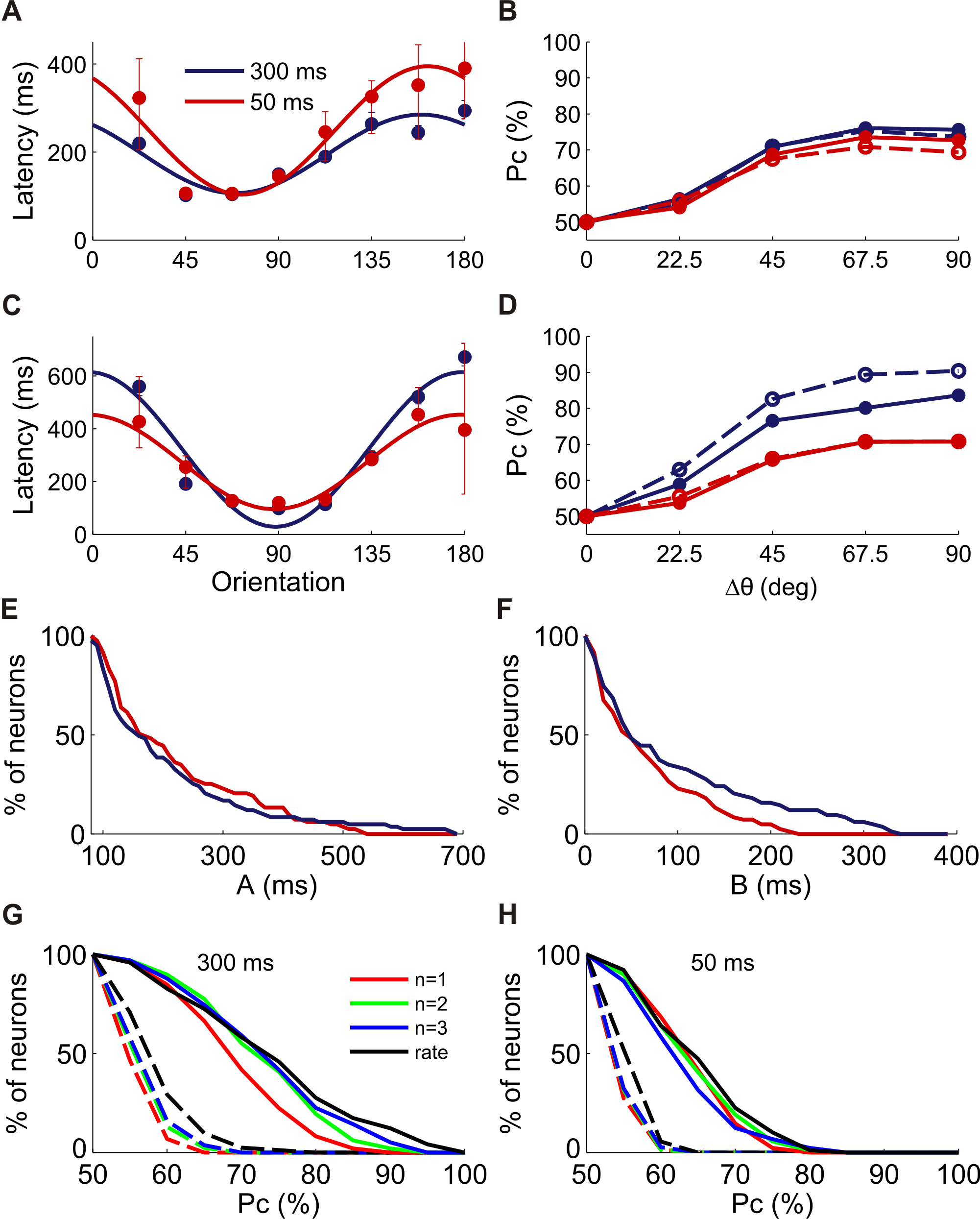

Supplement: Figure S2 — Latency tuning and orientation discrimination for static gratings. (A) First spike latency tuning curves for one cell. Different colors denote different stimulus durations (50 ms and 300 ms; see legend). (B) The corresponding neurometric curves for the same cell. For each stimulus duration, the solid curve corresponds to the first spike latency neurometric curve and the dashed curve to the conventional rate neurometric curve. (C)–(D) Same as (A)–(B) for a different cell. (E) Proportion of cells above a given level of the mean latency, A, for the two stimulus durations. (F) Proportion of cells above a given level of the modulation tuning, B, for the two stimulus durations. (G)–(H) Statistics of orientation discrimination. (G) Proportion of cells above a given performance level for the 300 ms stimulus. The dashed curves correspond to a 22.5° discrimination task and the solid curves to a 90° discrimination task. Different curves correspond to first spike latency (red), second spike latency (green), third spike latency (blue) and firing rate from the entire response (black). (H) Same as (G) for the 50 ms stimulus. The analyses were performed using dataset 6 in Table 1 (98 tuned cells for the 300 ms stimulus and 89 tuned cells for the 50 ms stimulus). (TIF) [file pcbi.1002536.s002.tif]

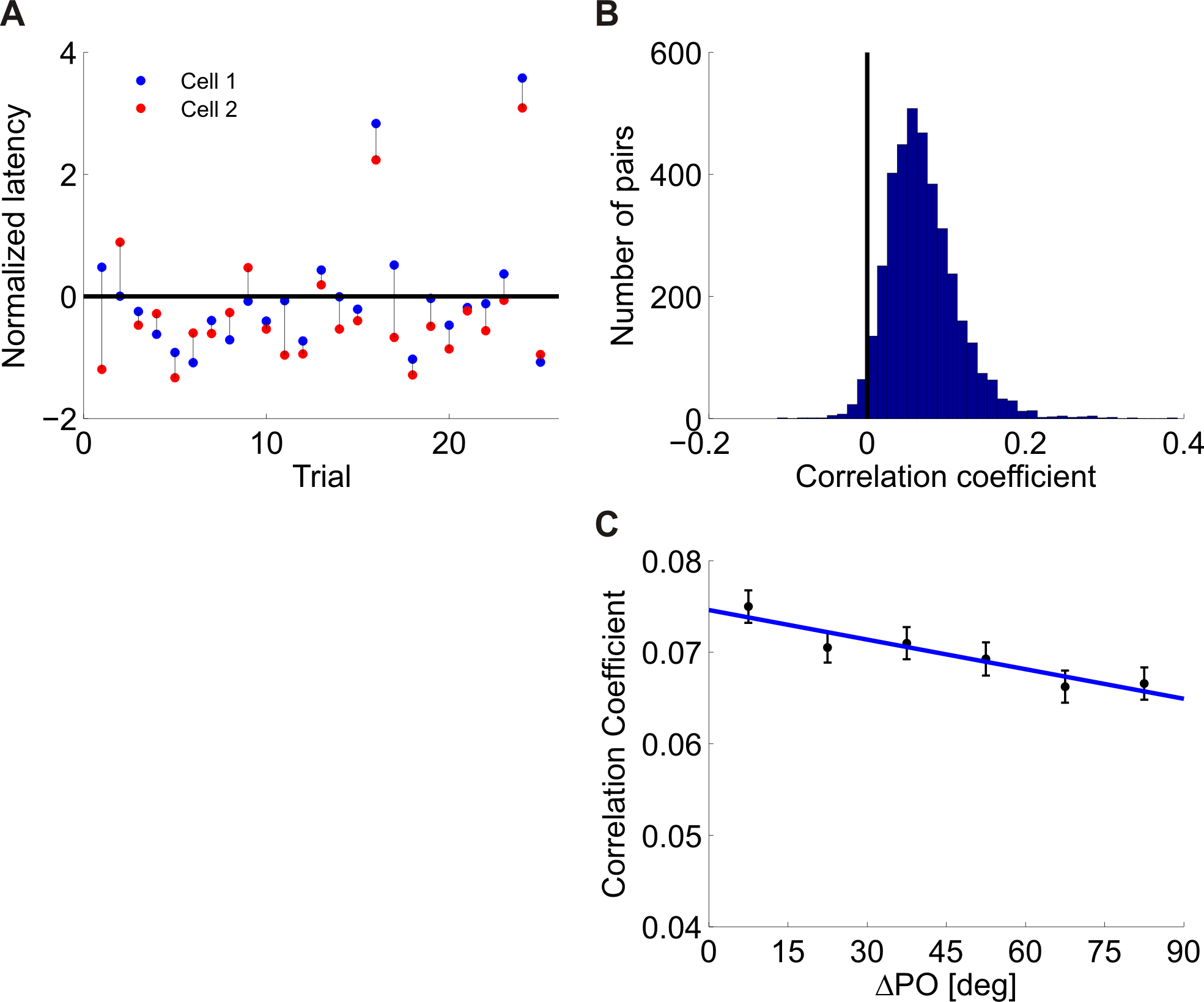

Supplement: Figure S3 — Pairwise correlations of response latencies among neurons. (A) Trial to trial fluctuations of first spike latencies for a pair of cells. For each neuron we calculated a normalized measure of its first spike latency by subtracting the mean latency and dividing by the standard deviation. These cells had similar latency preferred orientations, 139° and 136°, and their normalized first spike latencies are shown for the first 25 trials in which the stimulus orientation was 135°. The first spike latency of the two cells fluctuates from trial to trial around its mean. However, typically, when one cell fires sooner than its mean latency the other does as well, and similarly when the response is delayed, resulting in a positive correlation coefficient of 0.45. (B) Distribution of first spike latency correlation coefficients. For each pair of cells with latency tuning (B>15 ms) a correlation coefficient is calculated separately for each stimulus orientation. The mean of this distribution is 0.07 and its standard deviation is 0.04. (C) Dependence of correlations on the difference in preferred orientations (POs). The cell pairs were divided into six groups according to the difference between their preferred orientations, ΔPO. Each point represents the mean correlation coefficient for all pairs of neurons at a given range of ΔPO and the error bars represent the corresponding standard errors. The solid line represents the linear regression and its slope is −10−4±2•10−5 (deg-1). These results indicate that there is a very weak dependence of the first spike latency correlation on the difference between the latency preferred orientations of the cells. (TIF) [file pcbi.1002536.s003.tif]

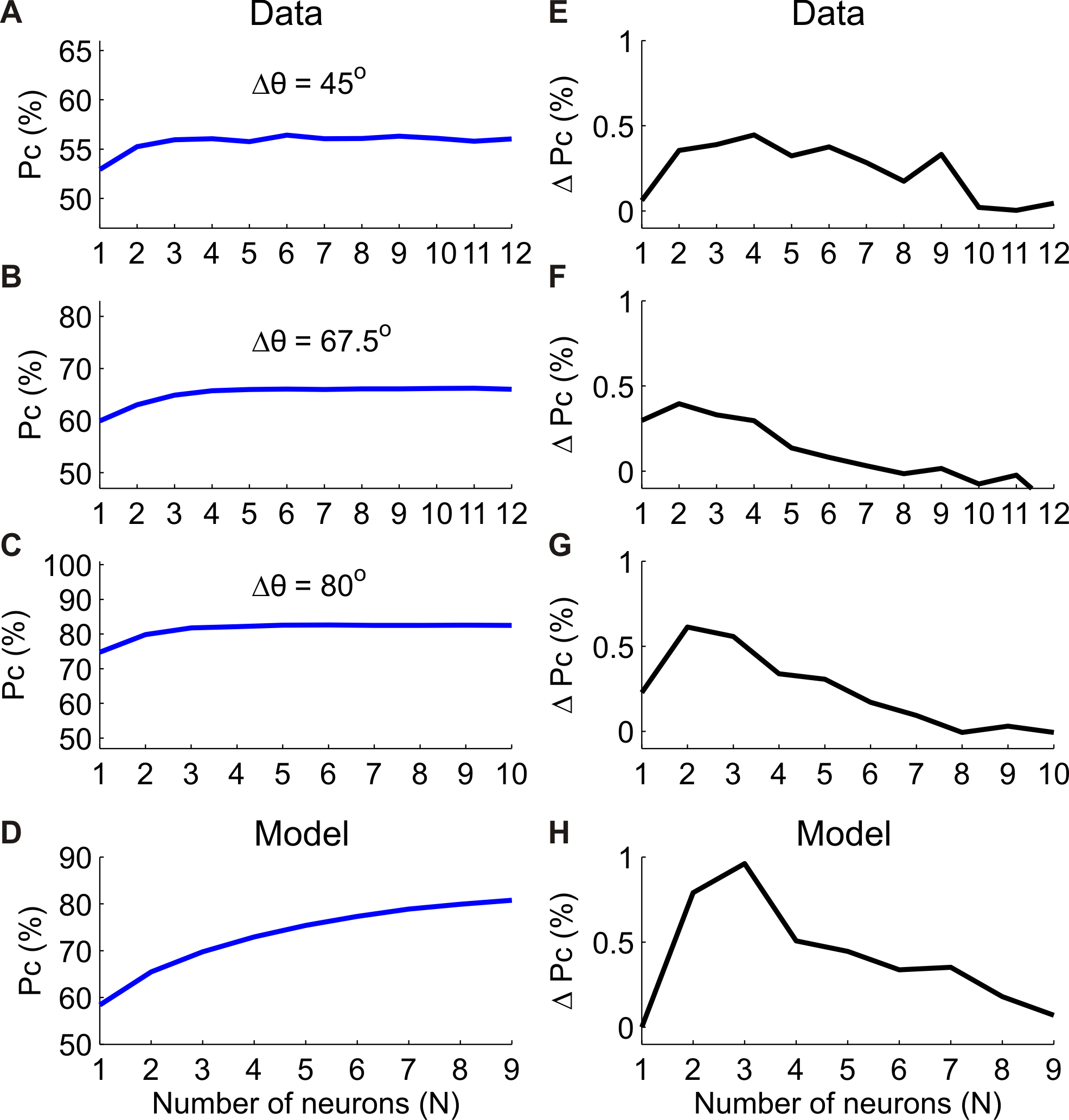

Supplement: Figure S4 — Effect of pairwise correlations on tWTA accuracy. Correlations in the trial to trial fluctuations of spike latencies can affect the utility of pooling information from groups of cells. To quantify the effect of correlations we compared the discrimination performance using the original simultaneous data to the performance using shuffled data with no correlations. In the shuffled data, the responses of different cells on a given trial were taken from different trials in the original data. The onset signal from the onset neurons was also shuffled among trials. For each shuffled version of the data we found the corresponding neurometric curve, and then averaged the result from 50 shuffles. (A–C) Probability of correct discrimination (Pc) as a function of population size (N) for two populations that differ in preferred orientation by 45° (A), 67.5° (B) and 80° (C) (same pairs of populations as in Figure 7). (D) Probability of correct discrimination in a model of two columns (see Text S1). (E–H) The effect of shuffling the responses of neurons in different trials. The curves show the difference in Pc between the original and the shuffled data (Pc(original)-Pc(shuffled)) for each pair of columns on the left. Although the performance is typically better in the original correlated data, the overall size of the effect is relatively small, on the order of 1%. For small populations, the correlations increase tWTA accuracy. However, as the population size increases, this difference decays to zero. The simplified model (H) captures the behavior of the data (see Text S1). (TIF) [file pcbi.1002536.s004.tif]

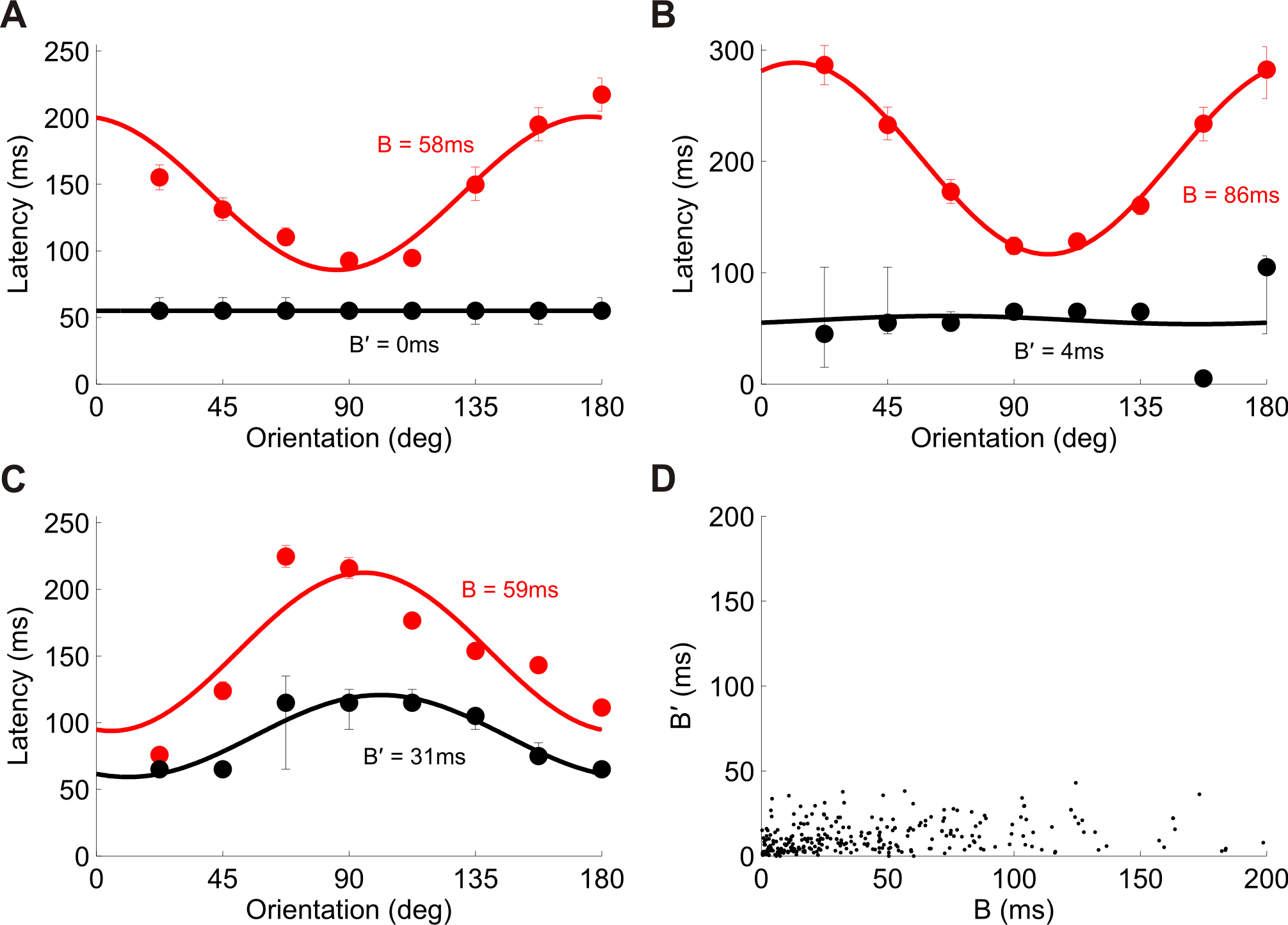

Supplement: Figure S5 — Effect of different definitions of response latency. Gawne et al. [26] recorded responses to oriented bars in V1 of behaving monkeys and reported that the effect of stimulus orientation on response latency is relatively weak. The difference with our results can be attributed to different definitions of response latency. Gawne et al. defined response latency to be the time at which the PSTH reaches its half peak (in cases where the peak was less than twice the spontaneous activity, latency was left undefined). We used the cumulative distribution function of first spike times and defined the first spike latency tuning curve as a level curve of this distribution. (A–C) First-spike latency tuning curves as computed by first spike (cumulative) distribution level curves (red) and the corresponding latency tuning curves for the same cells using the time at which the PSTH reaches half of its peak (black). (Lower and upper error bars for the halfmax definition were calculated using the times at which the PSTH reaches half the peak of the PSTH minus and plus its standard error of the mean). B denotes the modulation amplitude using our latency definition and B′ using the half max definition. For the cells in (A) and (B), the latency tuning curve using the half max definition (black) is relatively flat, whereas the first spike latency tuning curve using our definition (red) shows a strong modulation. For the cell in (C) both definitions show pronounced tuning. (D) Scatter plot of the modulation amplitudes, B and B′, for each cell. Notably, the tuning amplitudes using the halfmax definition are relatively small, whereas our definition results in many cells with significant modulation. (TIF) [file pcbi.1002536.s005.tif]

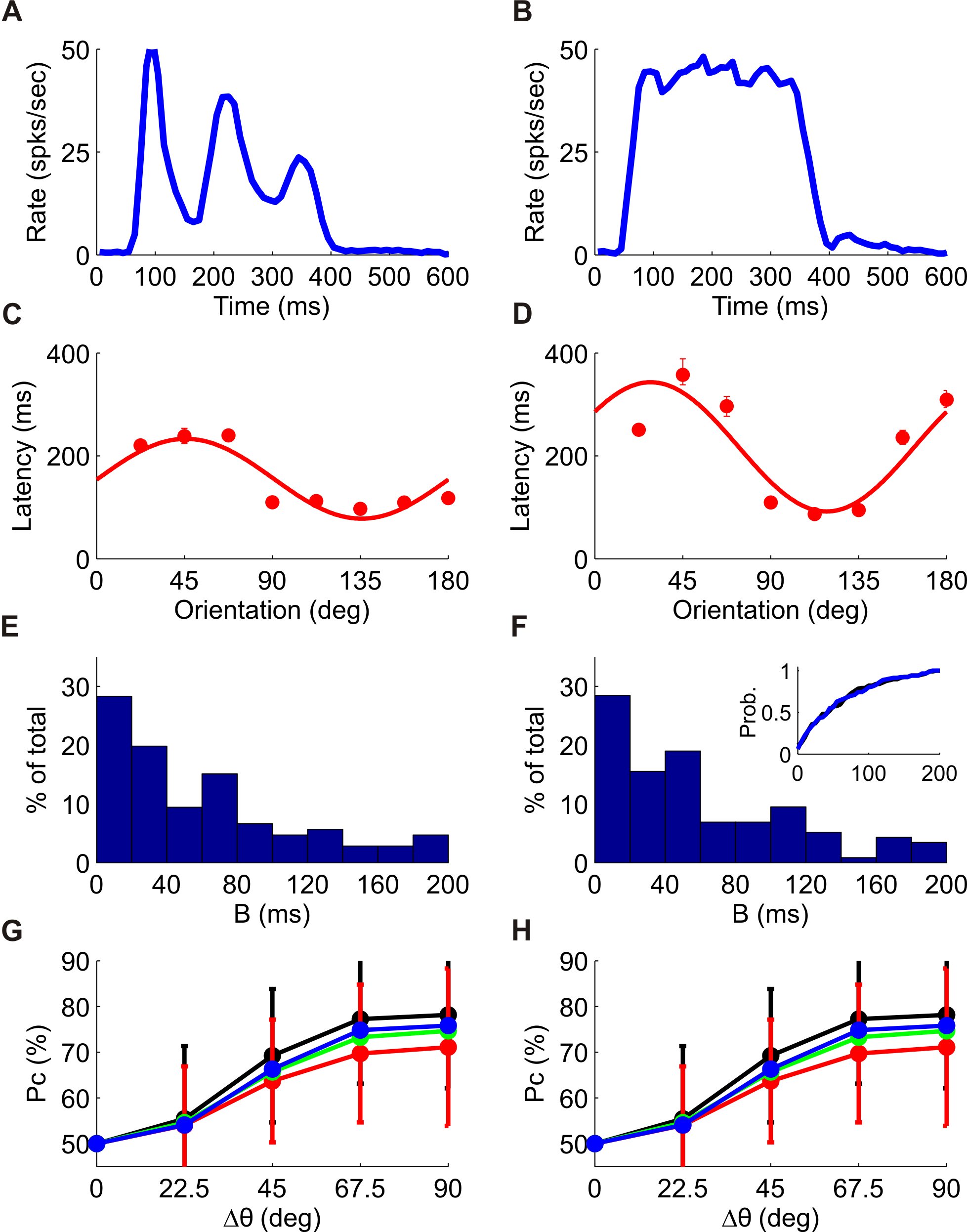

Supplement: Figure S6 — Effect of stimulus phase on spike latency tuning. The panels on the left side (A,C,E,G) depict results from a dataset in which all stimuli of the same orientation had identical initial phases; the panels on the right side (B,D,F,H) depict results from a dataset in which the initial phases were random. The two datasets were recorded in the same animal using the same electrode array. (A–B) PSTHs of two single cells. The stimuli were near the preferred orientation of the cells and the PSTH was constructed from 300 repetitions. For fixed initial phase, the PSTH is characterized by a periodic modulation whereas for random phases there is no such modulation. (C–D) First spike latency tuning curves of the neurons in (A) and (B). (E–F) Distribution of latency tuning modulation amplitude, B, in the two datasets. The substantial tuning of the response latency in the random phase dataset cannot be attributed to the stimulus initial phase. The inset in (F) shows the two cumulative distribution functions (black for the fixed phase dataset and blue for the random phase dataset). The similarity of the two distributions indicates that there is no significant difference between the orientation tuning levels of first spike latencies in the two datasets. (G–H) Mean neurometric curves using the first spike latency (red), second spike latency (green), third spike latency (blue) and the firing rate (black). Error bars represent the standard deviation and are shown only for the rate and the first spike latency neurometric curves. The fact that performance is similar indicates that our results reflect the tuning of the first spike latency to stimulus orientation and not the initial phase of the stimulus. (TIF) [file pcbi.1002536.s006.tif]
