# Supplementary material for: Fast Coding of Orientation in Primary Visual Cortex
Source: PLoS Comput Biol. 2012 Jun 14;8(6):e1002536. doi: 10.1371/journal.pcbi.1002536 (PMC3375217; doi:10.1371/journal.pcbi.1002536)
Supplement: Text S1 — A simple model for studying the effect of pairwise correlations on tWTA accuracy. (DOC) [file pcbi.1002536.s007.doc]

**A simple model for studying the effect of pairwise correlations on tWTA accuracy**

To gain insight into the effect of pairwise correlations among spike latencies on tWTA accuracy, we studied a simplified model of two orientation columns. The first spike latency of cell *k* in orientation column *i* (with preferred orientation $\theta_{i}$) is the sum of three contributions:

$$t_{k}^{(i)}=t_{0}+t^{(i)c}+t_{k}^{(i)u}$$

The first component,$t_{0}$, is a fluctuating time shift shared among all cells in both columns. Since tWTA is only sensitive to the order of firing and not to the absolute spike times, such trial to trial fluctuations that preserve firing order will not affect the tWTA decision. The second component, $t^{(i)c}$, is shared among all neurons in the same orientation column. Fluctuations in $t^{(i)c}$ generate uniform positive correlations within every orientation column and zero correlations between columns. Such correlations have been shown to limit the accuracy of the tWTA^10^. The last component, $t_{k}^{(i)u}$, is an independent component that contributes only to the variance of the single cell response. Since fluctuations in this component are independent, its effect is likely to decrease in large populations. All three components were drawn from distributions that correspond to a homogenous Poisson process. To one of the columns we also added a fixed delay, simulating the mean difference in first spike latencies between the two columns. In addition, each neuron had spontaneous activity, which was also modeled as a homogenous Poisson process. The parameters for the different components of the model were estimated from the data. We found that the uniform component, $t_{0}$, was much more dominant than the correlations that were not shared between the columns, $t^{(i)c}$(consistent with the weak dependence of the correlations on the preferred orientation difference, Fig. S3C).

We simulated this model and averaged tWTA accuracy over 10,000 trials, Fig. S4D. We then shuffled the simulated data and compared the results, Fig. S4H. As can be seen from the figure, the simplified model captures the behavior of the data. Although the uniform component does not affect the tWTA, when shuffled, it contributes to the variability of the single cell response; hence, for small populations tWTA performance in the shuffled data is inferior to the unshuffled data. As the population size increases, the effect of single cell variability decreases and the detrimental effect of the fluctuations that are not shared between columns increases; hence, tWTA accuracy in the unshuffled relative to the shuffled data decreases.
